# Evolutionary trend of the broad-snouted crocodile from the Eocene, Early Miocene and recent ones from Egypt

**DOI:** 10.1038/s41598-025-91167-w

**Published:** 2025-03-17

**Authors:** Eman S. El-Degwi, Mohamed K. AbdelGawad, Shaimaa E. Radwaan, Rania E. Sliem, Afifi Sileem, Salwa Ibrahim Abd Elhady

**Affiliations:** 1 Zoology Department, Faculty of Science, Benha University, Banha, Egypt; 2 Geology Department, Faculty of Science, Cairo University, Giza, Egypt; 3 Egyptian Geological Museum, Cairo, Egypt

**Keywords:** *Crocodylus niloticus*, *Rimasuchus lloydi*, Evolutionary trend, Broad snouted crocodiles, Ancestor, Egypt, Herpetology, Palaeontology

## Abstract

Skulls are a critical part of the crocodile through which we can distinguish between the different genera and species. Most of the crocodiles which previously studied from the Eocene–Oligocene to the Miocene times in Egypt were concerned with the identification of the genus and sometimes on the species without a detailed focusing on the evolution, comparing between them and trying to determine the ancestor or the closest species of them to the living crocodile in Egypt. The only known living species of *Crocodylus* in Egypt is *Crocodylus niloticus* which inhabits Lake Nasser in Aswan, southern of Egypt. From the Cenozoic era, broad snouted crocodiles diversity had been reported in Egypt. About 35 million years ago, through the Eocene epoch, the crocodilian fossils from Fayum provided evidence of the diversity of crocodile species including *Crocodylus articeps* and *Crocodylus megarhinus*. In addition to that, throughout the Early Miocene epoch, from about 18 million years ago, in Wadi Moghra Egypt crocodilian fossils demonstrate another diversity, extended to the first appearance of *Rimasuchus lloydi* which placed inside the *Osteolaeminae* later. By various measurements and carefully morphological examination of the different species recorded from Egypt, it was found that there are high levels of variation in morphology of the skulls including their dimensions, and the sutures shapes especially between premaxilla and maxilla ventrally and also between maxilla and palatine, as well as the extension of the maxillary ramus of the ectopterygoid. Using cluster analysis, it is proven that Eocene *Crocodylus* is the ancestor to all known broad snouted species recorded from Egypt since the Eocene time. The closest species to the Eocene specimen is the living *Crocodylus* *niloticus*. That in fact make that most of the broad snouted crocodiles in Egypt are endemic.

## Introduction

In evolutionary biology, morphology is crucial. Every contemporary group that still exists today preserves relics from the evolutionary path taken by its predecessors^1^.

The only archosaurian reptiles those remain are crocodilians^2,3^. The crocodilians first appeared in the Early Cretaceous, and they quickly spread throughout the world^4^. The order “crocodylia” refers to a group of large, semi-aquatic carnivores that found in nearly every kind of freshwater habitat in the tropics, subtropics, and some temperate regions^5,6^. One of the three major groups comprise modern crocodylians is the true crocodiles Crocodylidae (*Crocodylinae* and *Osteolaeminae*)^3^. According to^7,8^, the largest genus in the Order Crocodylia is *Crocodylus*.

The rostral shapes and proportions differ between taxa. Therefore, based on rostral proportions, crocodylians can be generally categorized into three groups: Longirostrine, Mesorostrine, and Brevirostrine^9^. Crocodilians are noted for their akinetic skull properties due to possessing a secondary palate^10,11^.

Fossils of mammals, selachians, teleosts, birds, and reptiles have been found in the Eocene–Oligocene rocks of the Fayum Province in Egypt. These rocks date from the Middle through the Upper Eocene and Lower Oligocene^12–14^. In these deposits, two crocodylians were collected from the Upper Eocene-Lower Oligocene of the Fayum Province *Crocodylus articeps* and *Crocodylus megarhinu*s^14,15^. Mook^16^ and Müller^17^ described an even more complete specimen of *C. megarhinus* which was acquired by the American Museum of Natural History (AMNH) in 1907. Additionally, Mook described an unassociated mandible (AMNH FARB 5095) that he stated belonged to *C. megarhinus* and was collected in 1909. In addition to the mentioned two crocodylians *Crocodylus* sp. is one of the taxa that have existed in the Fayum region since the Eocene.

Wadi Moghra is a fossil locality in the northern Sahara of Egypt that preserves a diversity of Early Miocene mammals and non-mammals. The non-mammals fossils are turtles, crocodiles, lizards, fishes, and avians^7,8,18–24^. *Crocodylus lloydi*, *Tomistoma downsoni*, and *Gavialis* species were found to be the majority of the crocodylians from the Moghra Formation listed in the works of Fourtau^25,26^ and El Khashab^7,27,28^. It was clearly that Wadi Moghra is the only place where *R. lloydi* is known to be certain Brochu and Storrs^29^. Fourtau^25^ initially identified *Rimasuchus*^30^ as *C. lloydi* in Moghra, Egypt. *C. lloydi* has been a part of multiple phylogenetic studies^31,32^, where they pointed out a closer relationship with extant African dwarf crocodile (*Osteolaemus*) than with *Crocodylus.* As a result of this^30^ classified the species under the new genus *Rimasuchus*. AbdelGawad^28^ clarified that the Moghra Crocodylian assemblages include four genera with highly morphological variety. *Tomistoma dowsoni*, *Rimasuchus lloydi*, *Euthecodon* sp. and *Crocodylus* sp. are the four genera. *Gavialis* species finally identified as *Euthecodon* sp.

Many different countries about forty two on the African continent comprise the Nile crocodile^33^. Shaker and El-bably^34^ display anatomical data on the bones of the *C. niloticus* skull which helps in understanding the explanation of X- ray images and surgical affection of the crocodile heads. The X- ray images were useful for recognizing the paranasal sinuses which their assistance to the morphological organization of the skull. The results spectacle a bony difference between the mammals, crocodile and also the birds. Also, another research on *Crocodylus*^35^, displays by using geometric morphometrics and geographic analysis. The author compares skulls of *C. niloticus* with other members of the genus *Crocodylus* in dorsal view to estimate interspecific and intraspecific differences. A *Crocodylus niloticus* osteological description is provided. Also, there is an analysis of model-based cluster and morphological clusters irrespective of other factors. The results prove the presence of a cryptic species complex. The largest living crocodylians with reported lengths up to 6 m is *Crocodylus niloticus* sensu lato^36^. There are differences within *C. niloticus s.l.* from the Congo River Basin where the sample was significantly difference from other regions including Nile River *C. niloticus s.l*.

This research aims to compare between the skull of the Egyptian fossils of broad-snouted crocodilian from the Eocene–Oligocene; Miocene and the living crocodile in Egypt (*Crocodylus niloticus*).

### Institutional abbreviations

Egyptian Geological Museum, Egypt (CGM); Cairo University Wadi Moghra collection and Cairo University Vertebrates paleontology Lab, Geology Department, Egypt (CUWM and CUVP); Duke Lemur Center, Division of Fossil Primates, Duke University, USA (DPC); Natural History Museum United Kingdom, London, United Kingdom (NHMUK); American Museum of Natural New York, U.S.A (AMNH); Peabody Museum of Natural History, Yale University New Haven, CT, U.S.A (YPM).

### Anatomical abbreviations

a, angular; ar, articular; as, alisphenoid; bo, basioccipital; bs, basisphenoid; cor, coronoid; cf., choanal fenestra; cqp, cranioquadrate passage; d, dentary; d1, dentary tooth postion 1; d4, dentary tooth postion 4; ect, ectopterygoid; emf, external mandibular fenestra; en, external naris; eo, exoccipital; eoa, external otic aperture; f, frontal; fae, foramen aereum ; fh, foramen hypoglossi; fim, foramen intermandibularis medius; fm, foramen magnum; gf, glenoid fossa; if, incisive foramen; imf, internal mandibular fenestra; itf, infratemporal fenestra; j, jugal; l, lacrimal; ldf, lacrimal duct foramen; lcf, lateral carotid foramen; lec, lateral eustachian canal; lsg, lateral squamosal groove; m, maxilla; mec, median Eustachian canal; mg, meckelian groove; mre, maxillary ramus of ectopterygoid; mt, maxillary teeth; n, nasal; o, orbit; oc, occipital condyle; oo, olfactory opening; ot, olfactory tract; otf, the orbito temporal foramen; pa., parietal; pal, palatine; pf, prefrontal; pfk; prefrontal knob, pm, premaxilla; pmt, primarily tooth; po, postorbital; pob; postorbital bar, pof, preotic foramen; pop, paroccipital process; pp, palatal process; pro; prootic, pt, pterygoid; ptf, post temporal fenestra; ptw, pterygoid wing; q, quadrate; qj, quadratojugal; rp, retroarticular process; sa, suprangular; sc, secondary choanae; soc, supraoccipital; sof, suborbital fenestra; sp, splenial; sq, squamosal; stf, supratemporal fenestra; sym, mandibular symphysis; v, vomer; vf, vagus foramen for IX, X,XI nerves; XII, exit foramen for 12th cranial nerve.

## Materials and methods

All the compared materials are cranial remains. The Eocene specimens included in this study are housed in CGM, NHMUK, AMNH and YPM VP Adams^14^ *Crocodylus articeps* upper jaw: NHMUK R 3322 (cast of holotype), CGM (C. 10036) (holotype); *Crocodylus articeps* lower jaw: NHMUK R 3323 (cast of holotype), NHMUK R 3324, NHMUK R 3105, CGM (C. 10065) (holotype); *Crocodylus megarhinus* upper jaw: NHMUK R 3327 (holotype), FARB AMNH 5061, YPM VP-058532; *Crocodylus megarhinus* lower jaw: NHMUK R 3328, FARB AMNH 5095; *Crocodylus* species upper jaw: CGM 84425. *Crocodylus* species lower jaw: NHMUK R 3104.

The Early Miocene specimens are preserved in the CGM^28^, DPC^28^, CUWM^28^ and NHMUK PVR. *Crocodylus* species upper jaw: CGM67106, CGM67107, CUWM90, CGM67123, DPC12548; *Rimasuchus lloydi* upper jaw: CGM67110, CGM67156, CGM73664, DPC6610, DPC6646, CGM67155, NHMUK PVR14154; *Rimasuchus lloydi* lower jaw: CGM67117, CGM67118, CGM67155.

All specimens were photographed in dorsal, ventral, palatal, lateral, occipital and posterior views with a scale placed.

The *C. niloticus* skull preparation included removing of different organs by using dissecting equipment. The cleaning and bleaching process happened by soaking in a metal container filled with boiling water with powdered detergent and powdered sodium carbonate. Then the running water was used to remove soft tissues manually from the skull. After that, it was soaked in hydrogen peroxide (H_2_O_2_) through which whiteness was achieved, then washed with water to remove any chemical excesses and finally the prepared specimens were left till became dry^34^.

The prepared *C. niloticus* skull was photographed and drawn from dorsal, ventral, lateral, and occipital views. The measurements of the *C. niloticus*, Eocene and Miocene specimens are taken and illustrated.

The biometric measurements of the investigated specimens were subjected to statistical analysis. The similarity and dissimilarity among the investigated taxa were processed via cluster algorithm analysis performed using a single linkage method and Jaccard similarity index. The PAST software, version 4.13, has been utilized to illustrate the retrieved dendrogram^37,38^.

## Results

### Systematic paleontology

Crocodyliformes^39^.

Mesoeucrocodylia^40^.

Neosuchia^41^.

Eusuchia^42^.

Crocodylia^43^.

Family Crocodylidae^44^.

**Fig. 1:**
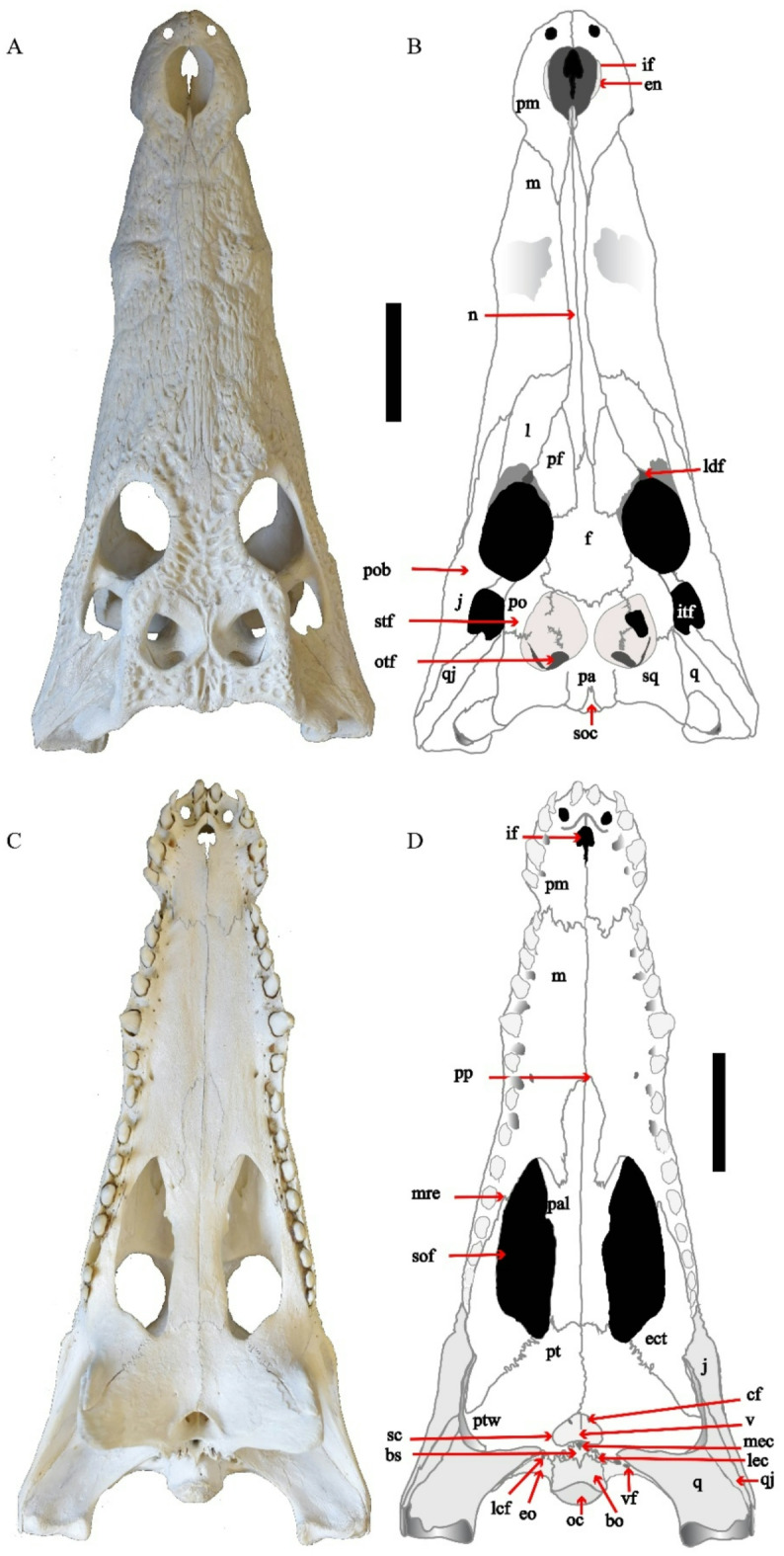
(**A**–**D**) CUVP001, *Crocodylus niloticus*, skull with a line drawing, (**A**,**B**) dorsal, (**C**,**D**) ventral. Scale bar equals 5 cm.

**Fig. 2:**
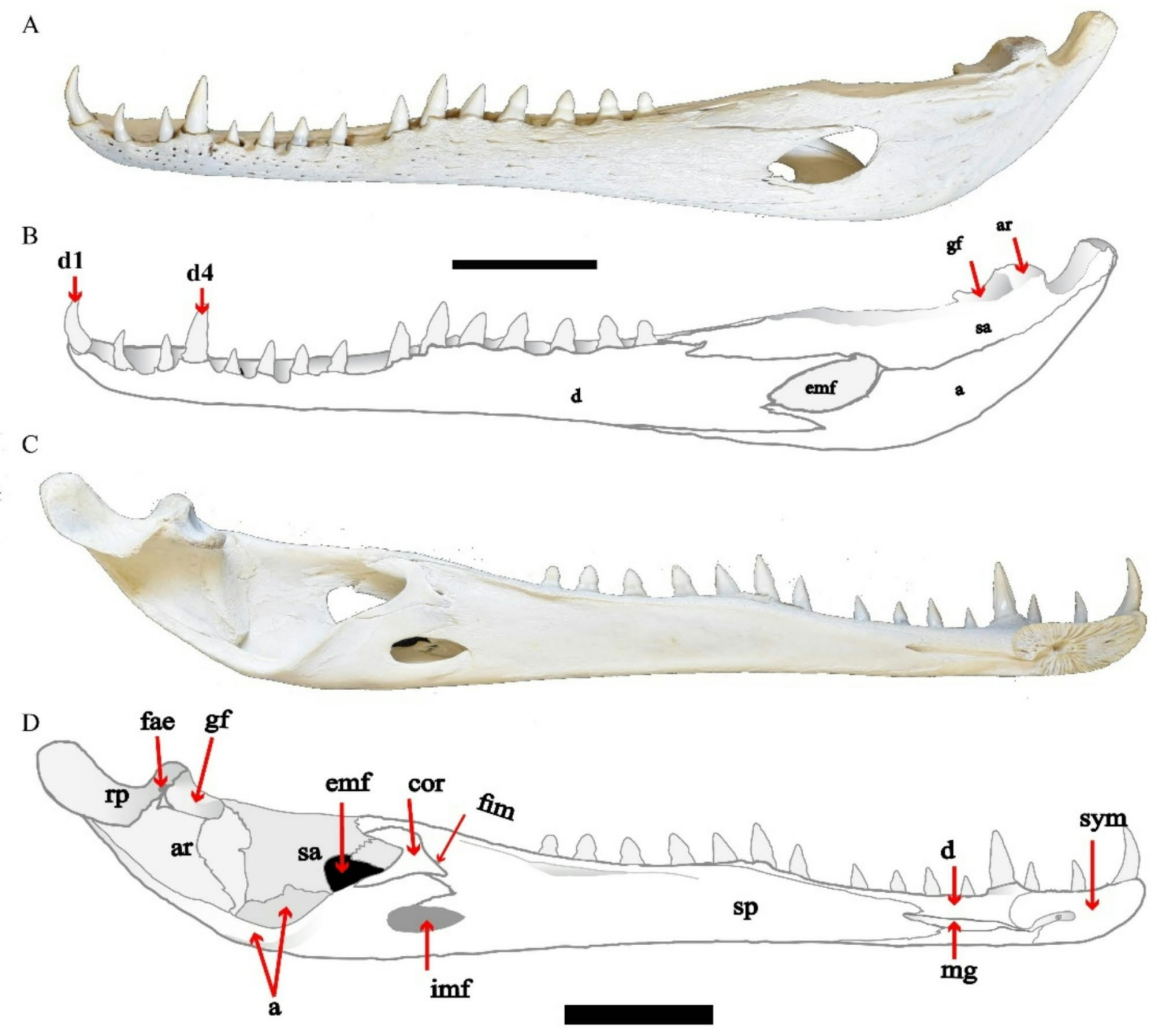
(**A**–**D**) CUVP001, *Crocodylus niloticus*, lower jaw with a line drawing, (**A**,**B**) labial, (**C**,**D**) lingual. Scale bar equals 5 cm.

**Fig. 3:**
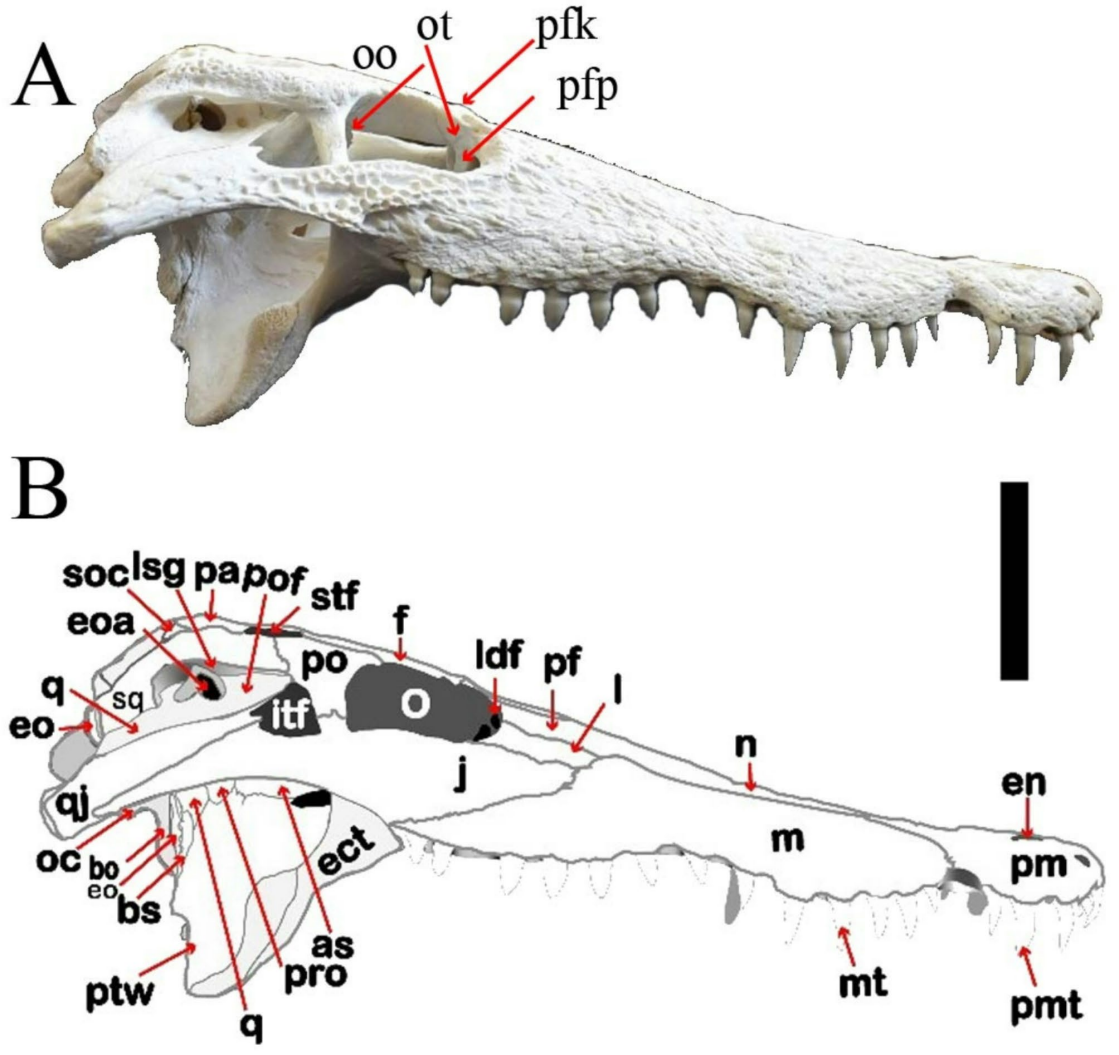
(**A**,**B**) is lateral view of CUVP001 *Crocodylus niloticus*, A, referred specimen; B, line drawing. Scale bar equals 5 cm.

**Fig. 4:**
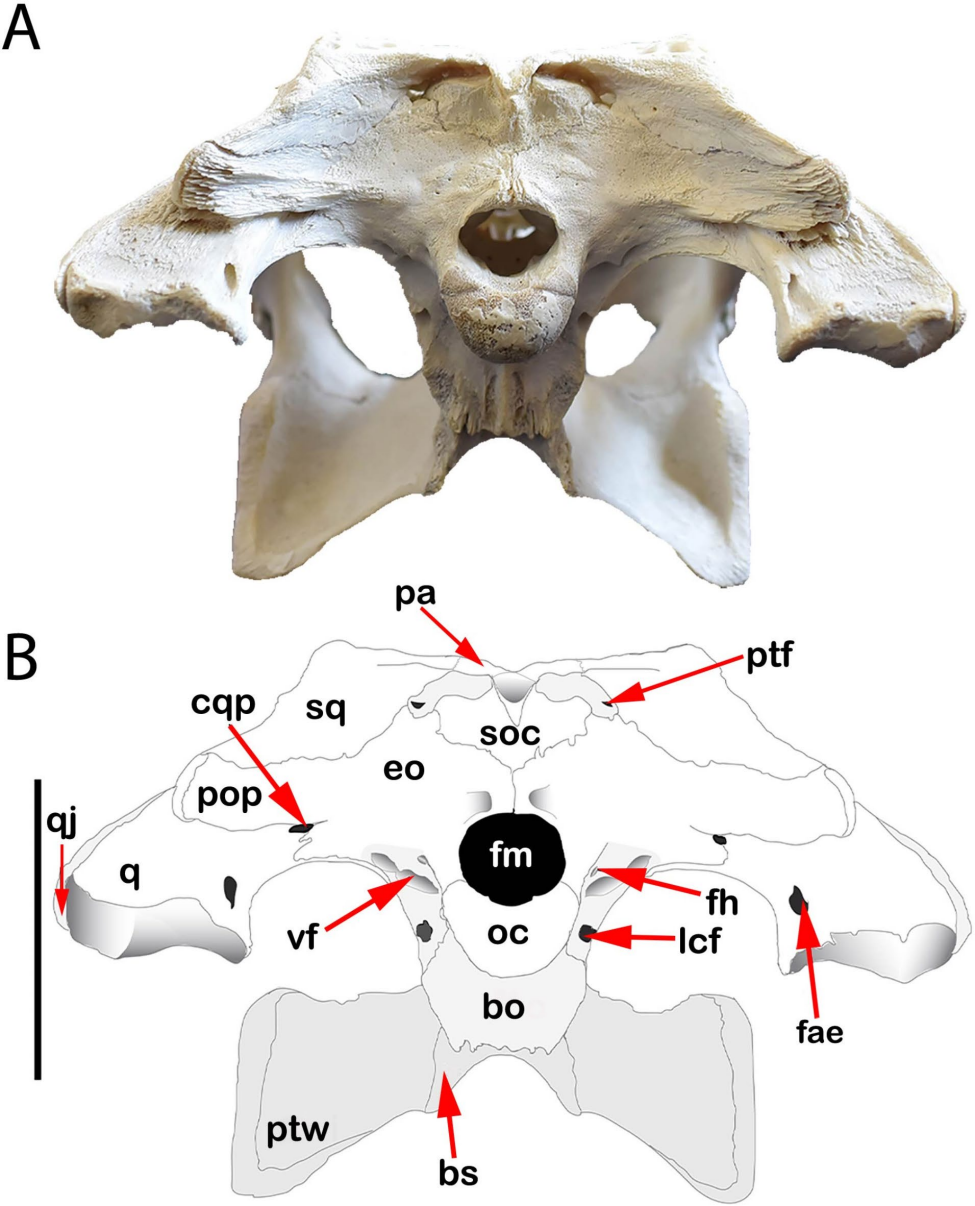
(**A**,**B**) is occipital view of CUVP001 *Crocodylus niloticus*, **A** referred specimen; **B** line drawing. Scale bar equals 5 cm.

The present study deals with the description of a complete skull and a mandible of *C. niloticus* (Figs. 1, 2, 3 and 4) and then compares it with skull descriptions of various broad-snouted crocodilian taxa from the Eocene and Miocene epochs.

The crocodile’s skull is referred to as a diapsid, or “two-arched,” reptile skull. A pair of large fenestrae are located at the dorsal wall of the skull, behind the large orbits; the supra and lateral temporal fenestrae. The skull of *C. niloticus* is characterized by a long, broad, and compressed dorsoventrally rostrum with long, sharp conical teeth. The rostrum bears numerous small openings called neurovascular foramina along the rostral alveolar borders. Also, small erratic pits in the dorsal surface become wider when they approach the orbits. The cranium is composed of the cranial and facial components.

### The facial components

Premaxilla is a paired bone that makes up the anterior portion of the rostrum. Dorsally, they are completely sub-circular. The premaxilla contacts with the nasal postero-medially and the maxilla postero-laterally then comes to a sharp point where the nasal, maxilla, and premaxilla intersect. The external naris lies dorsally in the premaxilla. There is a rugosity present lateral to the external naris that corresponds to the enlarged 4^th^ premaxillary alveolus. This rugosity expands the premaxilla laterally into the external naris, making it sub-circular in shape. Ventrally, the premaxilla intersects posteriorly with the maxilla only and terminates at the level of the second maxillary alveoli. The sutures between the premaxilla and maxilla are irregularly shaped almost making a W shape. There are five premaxillary conical teeth, different in the size of the alveoli, the second alveolus is smallest, whereas the fourth one is largest. Also, there are distinct gaps between the 1^st^ and 2^nd^ alveoli, the 3^rd^ and 4^th^ as well as the 4^th^ and 5^th^ ones.

Maxilla is paired, broad, flattened dorsoventrally (Fig. 1). It represents the largest bones in the snout. Dorsally, each one contacts with the nasal bones along their medial edge. The maxillae flare out laterally behind the premaxillae, narrow briefly posterior to the fifth alveolus, and then progressively expand posteriorly (Fig. 1). It contacts with the lacrimal bone posteromedially and the jugal bone laterally where the sutures between them are irregular. There is a large tuberosity “A rounded protuberance” toward the anterior portion of each maxilla above the large fifth maxillary tooth (Fig. 1). The maxillae are contact ventrally at the anterior midline but separated by the posterior palatines. There are fourteen maxillary teeth, the fifth maxillary tooth is the most robust and is circular, while the fourteenth is the smallest (Fig. 1). The fifth alveolus is positioned at the maximum concavity. Posteriorly the alveoli become smaller and more oval. Small occlusal pits are visible between the maxillary alveoli from the third one to the eighth maxillary alveoli anterior to the level of the suborbital fenestra for coinciding with the lower tooth row. The maxillae and dentaries have an undulating, concavo-convex lateral contour known as festooned Posteriorly, the maxillary tooth row is bordered by the anterior process of the ectopterygoid. The maxillary foramen for the palatine ramus of cranial nerve V is small. (Fig. 1).

Nasal is a pair of long and relatively narrow bones. They are located dorsomedially on the rostrum. Laterally, each nasal is contacted with the maxilla, lacrimal, and prefrontal, respectively, from the anterior to posterior (Fig. 3). Also, it is associated with the premaxilla anteriorly, the frontal posteriorly, and itself medially. Nasals narrow anteriorly where they intersect with the premaxillae and send a short process into the external naris called anterior processus nasalis. The posterior ends form acute points separated by the anterior process of the frontal. The nasals rejoined medially at the level of the anterior margin of the 10^th^ alveolus. The suture is obvious along the shared medial margin of the nasals, but it diverged slightly as they entered the distinctly pear-shaped external naris. The nasal breadth narrows anteriorly near the external naris and posteriorly between the frontals. At the approximate level of the most anterior edge of the maxillae, the nasals gradually widen and they gradually narrow at the level of the 6th maxillary alveolus while widen again, reaching their greatest width at the level of the 9th maxillary alveolus at the most anterior prefrontal margin level.

Lacrimal lies anterior and lateral to the prefrontals. In the dorsal view, the medial surface of each lacrimal contacts with the nasal anteriorly and the prefrontal posteriorly. It contacts with the maxilla antero-laterally and the jugal postero-laterally (Fig. 3). The posterior margin of the lacrimal makes up the most anterior margin of the orbit and bears a lacrimal duct (Fig. 1). The maxilla-lacrimal suture is irregular.

Prefrontal is approximately triangular in dorsal view (Fig. 1). The anterior portion of each prefrontal is wedge-shaped and projects anteriorly. It contacts with the frontal postero-medially, the lacrimal laterally, and the nasal rostrally. Prefrontals comprise the antero-medial margins of the orbit. The medial prefrontal-frontal suture is straight but curves sharply posteriorly to intersect with the orbit (Fig. 1). Ventrally, prefrontals send robust vertical processes called the prefrontal pillars that contact with the dorsal surfaces of the pterygoids and palatines (Fig. 3). This process supports the secondary ossified palate. The prefrontal pillars do not contact with each other.

Jugal bones are paired bones. They make up the majority of the ventral margins of both the lateral temporal fenestrae and orbits. Dorsally, it is surrounded by the maxilla anteriorly, lacrimal antero-medially, and the quadratojugal posteriorly (Fig. 1). The jugal-lacrimal suture is irregular. The ventral margin of the postorbital bar is inset from the lateral jugal surface. The jugal narrows gradually towards the posterior, terminating in a sharp point just anterior to the end of the quadratojugal. In the ventral view, the jugal bone meets the ectopterygoid dorsoventrally.

Quadratojugal is a pair of elongated bones. This bone is situated between the jugal antero-laterally and the quadrate postero-medially. The quadratojugals comprise the posterior margin of the lateral temporal fenestrae and send within them spine-like processes termed quadratojugal spines (Fig. 1). The broad contact with the posterior part of the jugal is extremely elongate and postero-ventrally oriented.

Quadrate is large, found at the postero-lateral wall of the skull. The distal borders of the quadrates make up hemicondyles that articulate with the articular bones in mandibles. In dorsal view, quadrate is located between the temporal and quadratojugal bones (Fig. 1). The anterior portion of the quadrate is covered by the squamosal and in the occipital region the quadrate is overlain by the exoccipital medially toward the anterior side. Dorsally the quadrate contains on the medial portion foramen aereum (Figs. 1 and 4a and b). Quadrates form the floor and posterior margin of the external otic aperture.

Postorbital forms most of the posterior margin of the orbit as well as the anteromedial margins of the lateral-temporal fenestrae (Fig. 1). Medially, postorbitals form sutures with the frontal and parietals, excluding the frontal from connecting with the supratemporal fenestrae. The suture between the postorbital and squamosal is oriented ventrally to the skull table (Fig. 1). The postorbital has a descending process that contacts with the ascending process of the jugal and ectopterygoid to form the postorbital bar (Fig. 3).

Palatine is a long, paired bone and meets at the midline. Each palatine contacts with the maxilla anterio-laterally, and the pterygoid posteriorly (Fig. 1). The sutures with the pterygoids are very irregular and transverse with a convex curve; they do not occur at the most posterior margin of the suborbital fenestra. Its lateral margin makes up the anteromedial edge of the palatal fenestra. The palatine has an elongated, anterior process that extends to approximately the level of the 6^th^ maxillary tooth position (Fig. 1). The palatine-maxilla suture intersects with the suborbital fenestrae at the beginning of 9^th^ maxillary alveolus which is situated at the most anterior end of the suborbital fenestrae.

Ectopterygoid is a paired, tripartite one. Ventrally, it contacts with each of the maxilla anteriorly and with the pterygoid lateromedially and postero-laterally in addition to forming the lateral boundary of the suborbital fenestra (Fig. 1). Posterior to the suborbital fenestra, ectopterygoids are oriented postero-ventrally. Both ectopterygoids extend to the border of the 10^th^ maxillary alveoli so, it is parallel to the last five maxillary alveoli. The maxillary ramus of ectopterygoid is forked. The ascending process of the ectopterygoid articulates with the jugal and forms the lateral part of the lower half of the postorbital bar (Fig. 3).

Pterygoid is a paired, broad bone. In the ventral view, it is articulated anteriorly with the palatine (Fig. 1). The pterygoids meet at the midline and contact with the latero-sphenoid. In connection with the palatines a narrow wedge of pterygoid creates a flared-out structure, intersecting the suborbital fenestra medially, so it makes the part of postero-medial margin of the palatal fenestra and meets the basisphenoid postero-ventrally. It consists of a body and wing; the body bears an unpaired opening along the midline, called the secondary choanae. Latero-ventrally the pterygoids form sutures with the ectopterygoids. Directly posterior to the intersection of the pterygoid and ectopterygoid there are two shallow recesses separated by a narrow ridge. Near the midline, dorsal to the internal choana, a thin lamina extends dorsally on each side, forming a V-shaped saddle, where the basisphenoid sits ventrally to the median eustachian foramen. The pterygoids completely enclose the duct behind the suborbital fenestrae before exiting at the internal choanae. Wings are dorso-ventrally thickened and form a buttress at the articular surface with the ectopterygoid, so it meets the ectopterygoid antero-laterally.

Vomer is a single bone that divides the secondary choana into two cavities, or subfossae called the choanal fenestra (Fig. 1).

### The cranial bones

The occipital bones constitute the most posterior part of the skull. They are composed of a completely fused four bones enclosing a foramen magnum; the supraoccipital, basioccipital, and exoccipital on each side.

The supraoccipital exists in heart shape in the occipital surface dorsomedially. The supraoccipital is exposed on the dorsal surface of the most posterior part of skull as a very narrow triangular wedge, separating the posterior margin of the parietal (Fig. 1). The ventral margins of supraoccipital come together in a V- shape to meet the exoccipitals in occipital view so it contacts with the exoccipitals laterally and ventrally (Fig. 4). In the occipital view, the supraoccipital becomes inward from the edge of the most posterior part of the skull and forms the medial margins of the post- temporal fenestra (Fig. 4).

The exoccipitals meet at the midline and dorsal to the foramen magnum, separating the supraoccipital from the foramen magnum (Fig. 4). They are extended laterally forming the paraoccipital processes. This process is bordered dorsally and anteriorly by the squamosal and ventrally by the quadrate (Fig. 4). The exoccipitals are sutured with the squamosal bone dorso-laterally and the supraoccipital bone dorso-medially. The exoccipital meets the basioccipital ventrally. They form the dorsal and lateral margins of the foramen magnum. Also, it is surrounded ventrally by the basioccipital. Also, it is surrounded ventrally by the basioccipital. Within the braincase, exoccipital forms the most posterior portion of the roof of the cranial cavity. It also contributes greatly to the posterior wall of the otic capsule.

The basioccipital makes up the most ventral portion of the most posterior part of the skull. It contacts with the exoccipital dorso-laterally in the occipital view (Fig. 4). It had mainly a single spherical shape occipital condyle which articulated with the condyloid fossa of the atlas vertebra forming the atlanto-occipital joint (Fig. 4). Dorsal to the occipital condyle it makes the ventral boundary of the foramen magnum which represented the posterior floor of the cranial cavity. It contacts with the basisphenoid along its posterior margin (Fig. 4).

Prootic is visible as a small crescent shaped bone. The prootics bound the trigeminal foramina posteriorly and contact with the laterosphenoids anteriorly, while the quadrates cover them medially (Fig. 3).

The laterosphenoid lies ventral to the parietal. It is overlain in part by the postorbital along its lateral edge (Fig. 3). Ventrally these bones contacts with the pterygoid, quadrate and prootic posteriorly where they make up the anterior portion of the braincase and meet at the midline. Anteriorly they contact with the main body of the frontal since they form the ventral opening for the olfactory tract (Fig. 3). Ventral to the olfactory opening they form the opening for the optic nerve. Anteriorly, it bounds a deep oval-shaped opening of the trigeminal foramen.

The basisphenoid is bounded by the pterygoid anteriorly and the basioccipital posteriorly. Also, it articulates laterally with the quadrate and exoccipital. It is visible ventrally, just posterior to the secondary choana (Fig. 1). Between the basioccipital and basisphenoid bones behind the choana there a singular opening of the median Eustachian tube and the paired openings of lateral Eustachian canals. (Fig. 1).

Frontal is a singular bone. Dorsally, it is bordered anteriorly by the nasals, antero-medially by the prefrontals, postero-laterally by the postorbitals, and posteriorly by the parietal (Figs. 1 and 3). The prefrontal-frontal suture is straight temporarily before curving sharply to intersect with the orbit. The frontal does not participate in the formation of supratemporal fenestrae. A deep and well-defined olfactory canal is formed by descending processes of the frontal.

Parietal contacts with the frontal bone in the dorsal view, thus the fronto-parietal contact is concave anteriorly, it bounds to the squamosal postero-laterally. Also, it has a narrow contact with each of the postorbitals antero-laterally and the supraoccipital bones posteriorly (Fig. 1). The ventral margin of the parietal makes up a portion of the post-temporal fenestrae. Most of the lateral margins of the parietal comprise the supratemporal fenestrae. The parietal expands to make up most of the interior portion of the supratemporal fenestra. The interfenestral bar is flat dorsally and narrow.

Squamosal is paired and makes up the posterior and lateral portion of the skull table. It forms the postero-lateral margin of the supratemporal fenestra. It is sutured to the postorbital antero-dorsally, parietal laterally, and the exoccipital posteriorly. In the occipital view, the squamosal participates in the lateral margin of the post-temporal fenestra (Fig. 4). This bone connects with the lateral temporal fenestrae anteriorly. The strongly developed descending process of the squamosal postero-dorsally overlies the quadrate and laterally overlaps the exoccipital (Fig. 3). The most posterior part of the ventral edge of the temporal bone curved over the otic recess (Fig. 3) and contacted with the quadrate bone. The squamosal is expanded laterally and postero-laterally where it forms the dorsal roof and a part of the posterior wall of the external otic aperture.

### Foramina and fenestrae

External naris is a large pear-shaped nose orifice situated dorsally on the premaxillae. It is bounded anteriorly and laterally by the premaxillae and posteriorly by nasal processes. About 2.5 cm separates the external naris from the tip of the snout. The length of the external naris is greater than its width, and the narial chamber is rather deep (Fig. 1).

Foramen for the first dentary tooth is a circular foramen in the shape dorso-ventrally. It presents on the premaxilla anterolaterally to the external naris and between alveoli 1–2 from the ventral view.

The incisive foramen is another opening on the premaxillae posterior to the 1^st^ premaxillary alveoli, smaller than the external naris. This foramen has a pointed edge anteriorly and its posterior margin is separated into three lobes, the lateral two are short and rounded, while the medial one is narrow, pointed, and longer than the other two lobes (Fig. 1).

Orbit is surrounded by seven bones, the prefrontal, frontal, lacrimal, jugal, and postorbital (Figs. 1 and 3). The anterior wall of the orbit is bounded by the lacrimal bone while its medial wall is formed by the prefrontal and frontal. The jugal is located near the orbit’s border dorsoventrally at the margin of the orbit. The posterolateral orbital margin is formed by the postorbital bone. The postorbital bar forms the posterior margin of the orbit. The orbits are wider than supratemporal and lateral temporal fenestrae. Each orbit converges to a rounded point anteriorly (Fig. 1). Along the anterior margin of the orbit, within the prefrontal-lacrimal suture lies the lacrimal foramen. There is a palpebral ridge along the medial portion of the orbit that expands dorsally.

Supratemporal fenestrae is relatively sub circular in the shape. Dorsally, the supratemporal fenestrae are bounded laterally by the squamosal-postorbital suture, anteriorly by the postorbital-parietal, and posteriorly by the squamosal-parietal sutures (Fig. 1). The supratemporal fenestra did not connect with the frontal due to its medial contact with the parietal and postorbital anteriorly. The flat and narrow interfenestral bar creates the medial walls of the supra-temporal fenestrae. The interfenestral bar is formed by the parietals and it is expanding ventrally (Fig. 1).

Lateral temporal fenestrae: Infratemporal fenestra is triangular and can be seen the process from quadratojugal. The infratemporal fenestrae are medium in size, being slightly larger than the supratemporal one while it is smaller than the orbit. The fenestra is bounded by the quadratojugal and quadrate posteriorly and the postorbital bar anteriorly, the squamosal medially, and the jugal laterally (Figs. 1 and 3).

The external otic aperture (Recessa otica externa) is a deep opening located ventral to the overhang formed by the squamosal’s lateral border. The squamosal forms the external otic aperture’s dorsal roof and a portion of its posterior wall, while the quadrate forms the external otic aperture’s floor and part of the posterior boundary. The preotic foramen is located anterior to the external otic aperture (Fig. 3).

Olfactory opening is the opening for the olfactory tract which is formed by the connection between anterior part of laterosphenoids and ventral main body of frontal (Fig. 3).

Optic foramen is the opening formed by the laterosphenoids medially ventral to the olfactory opening. This foramen creates for the optic nerve.

The orbito-temporal foramen is a small opening. It is situated in the posterior wall of the supra-temporal fenestra. It is surrounded dorsally by the squamosal, quadrate, and ventrally by parietal (Fig. 1).

Posttemporal fenestrae are small, crescent-shaped openings on the occipital region’s surface (Fig. 4). The post-temporal fenestra is bounded by the squamosal dorso-laterally, the exoccipital ventro-laterally and by the supra-occipital ventro-medially and dorso-medially. The occipital veins pass through their ventral margin. They also contribute to the anterodorsal portion of the otic capsule.

Palatal fenestrae (the suborbital fenestrae) are elongated oval fenestrae in the shape. They have a rounded end anteriorly. Palatal fenestrae is bounded anteriorly by the maxilla, postero-laterally by the ectopterygoid, posteriorly by the pterygoid and medially by palatines. These fenestrae extend from the beginning of the ninth maxillary teeth to the level posterior the fourteenth ones. Much above the anterior border of the suborbital fenestra is the anterior palatal process of the palatines (Fig. 1).

Trigeminal foramen (foramen ovale) is an oval, deep opening. This foramen lies on the skull’s lateral surface. The trigeminal foramen for the passage of the trigeminal nerve V. The quadrate expands forming the ventral margin and part of the posterior margin of it. It is surrounded posteriorly by the prootic and anteriorly, by the laterosphenoid (alisphenoid).

Secondary choana lies ventrally at the skull’s posteromedial portion. Pterygoids completely enclosed it (Fig. 1). It is divided by the vomer bone into two cavities called choanal fenestra.

Median and lateral Eustachian canals lie in the depression between the basioccipital and basisphenoid bones ventrally. The lateral Eustachian canals are paired openings and the median Eustachian tube (foramen intertympanicum) is the singular opening. The openings of lateral Eustachian canals are presented posterolateral to the median one. The median eustachian opening is almost enclosed completely by the basisphenoid, although the basioccipital forms a small part of its posterior margin. The lateral eustachian openings connect with the basisphenoid anteromedially, anterolaterally and with the basioccipital posterolaterally (Fig. 1).

Foramen aereum is located on the quadrate’s medial dorsal surface at a corresponding opening on the articular of the mandible (Fig. 4). It contributes to skull pneumatization (siphonium).

Foramen magnum is a circular opening in the center of the occipital surface then lies above the occipital condyle (Fig. 4). Laterally to the foramen magnum there are four foramina. The medial and small one is the foramen hypoglossal for the passage of the XII cranial nerve (Hypoglossal nerve), which lies directly lateral to the foramen magnum (Fig. 4). Ventrally and slightly lateral to the foramen hypoglossal and just dorsal to the basi-occipital there is a foramen which is a much larger foramen “Lateral carotid foramen” which acts as the passage for the internal carotid artery (Fig. 4). Lateral and near the base of the occipital condyle, the otoccipital is pierced by two foramina” The larger one was the foramen vagus for the passage of the IX, X, and XI nerves as well as the jugular vein. The dorso-lateral foramen (The cranioquadrate passage) is a foramen for each the facial nerve (VII) and the cranioquadrate. The cranioquadrate passage opens laterally beneath the paroccipital process.

### Lower jaw

Mandible is formed by the two halves fused together anteriorly by the median mandibular symphysis via dentary bone. Each half of the mandible was composed of six fused bones; articular, angular, supra-angular, coronoid, splenial, and dentary. The mandible has an oval-shaped external and internal mandibular fenestra (Fig. 2).

Articular is a small bone at the most posterior end of the mandible and bears the glenoid fossa which is the quadrate’s articulation surface. The articular articulates medially with the surangular and ventrally with angular (Fig. 2). On the medial surface of the articular where the retroarticular process meets the glenoid cavity which is the foramen aereum. The foramen aereum occurs at the inner margin of the base of the retroarticular process.

Surangular is a dorso-ventrally broad and long bone forming the dorsal mandibular margin. The surangular articulates medially with the articular and forms the lateral wall of the glenoid fossa and angular ventrally. It rests between the dentary and splenial dorsally and projects forward forming a part of the dorsal and posterior margins of the external mandibular fenestra. Its anterior contacts with the dentary become somewhat sinuous and forms two anterior processes of unequal length (Fig. 2). A postero-dorsal process of the dentary narrowly separates the surangular from the antero-dorsal margin of the external mandibular fenestra. External mandibular fenestra is bounded by the surangular postero-dorsally, dentary antero-dorsally, and angular ventrally. The surangular–angular suture intersects the fenestra at its most posterior edge and not along its postero-ventral margin.

Angular forms the ventral margin of the posterior part of the mandible. It forms a part of the ventral and posterior margin of the external mandibular fenestra (Fig. 2). Posterior to the mandibular fenestra, the angular contacts with the surangular dorsally and dentary anteriorly. Deep pits cover most of the ventral surface of it.

Dentary is the largest bone in the mandible which contains 15 sharp conical lower teeth at its alveolar border (Fig. 2). The 4th dentary alveolus is enlarged due to the 4^th^ dentary tooth is the largest one. Posterior to the last alveolus, the dentary is overlain by the surangular. Anteriorly, the dentaries meet at the midline and form the mandibular symphysis. The symphysis extends from the 1^st^ dentary alveolus to the beginning of the 4^th^ one. The dentary comes into contact with the splenial at the level of the 6^th^ dentary alveolus. The dentary extends posteriorly to form the anterior and dorsal borders of the external mandibular fenestra. The external suture between the dentary and angular is ‘V’-shaped with a small process of the dentary underlying the level of the external mandibular fenestra but separated from it by a small flange of the angular.

Splenial exists in the inner surface of the mandible. The paired splenial is a long bone that contacts with the dentary dorsally and ventrally along its medial margins (Fig. 2). It contacts with the surangular postero-dorsally. The dorso-medial margin of the splenial reaches to the coronoid, and the ventro-medial margin contacts with angular.

Coronoid occupies the inner surface of the mandible (Fig. 2). The coronoid is relatively small. It contacts with both of the splenial anteriorly, the angular ventrally, and the surangular dorsally. It forms the posterior and ventral surface of the foramen intermandibularis medius (Fig. 2). The posterior surface is sharply concave.

The foramen aereum is located at the mandibular lingual edge in the base of the retroarticular process (Fig. 2).

The external mandibular Fenestra is enclosed by the supra-angular postero-dorsally, the angular ventrally, and the dentary antero-dorsally (Fig. 2). The suture between surangular and angular intersects the external mandibular fenestra at its most posterior margin.

The internal mandibular fenestra is surrounded by the splenial.

## Discussion

The present study deals with the cranial description of broad snouted crocodile *C. niloticus* from Egypt and comparing with the Eocene and Miocene Crocodiles in Egypt and pointing out the similarities and differences.

**Fig. 5:**
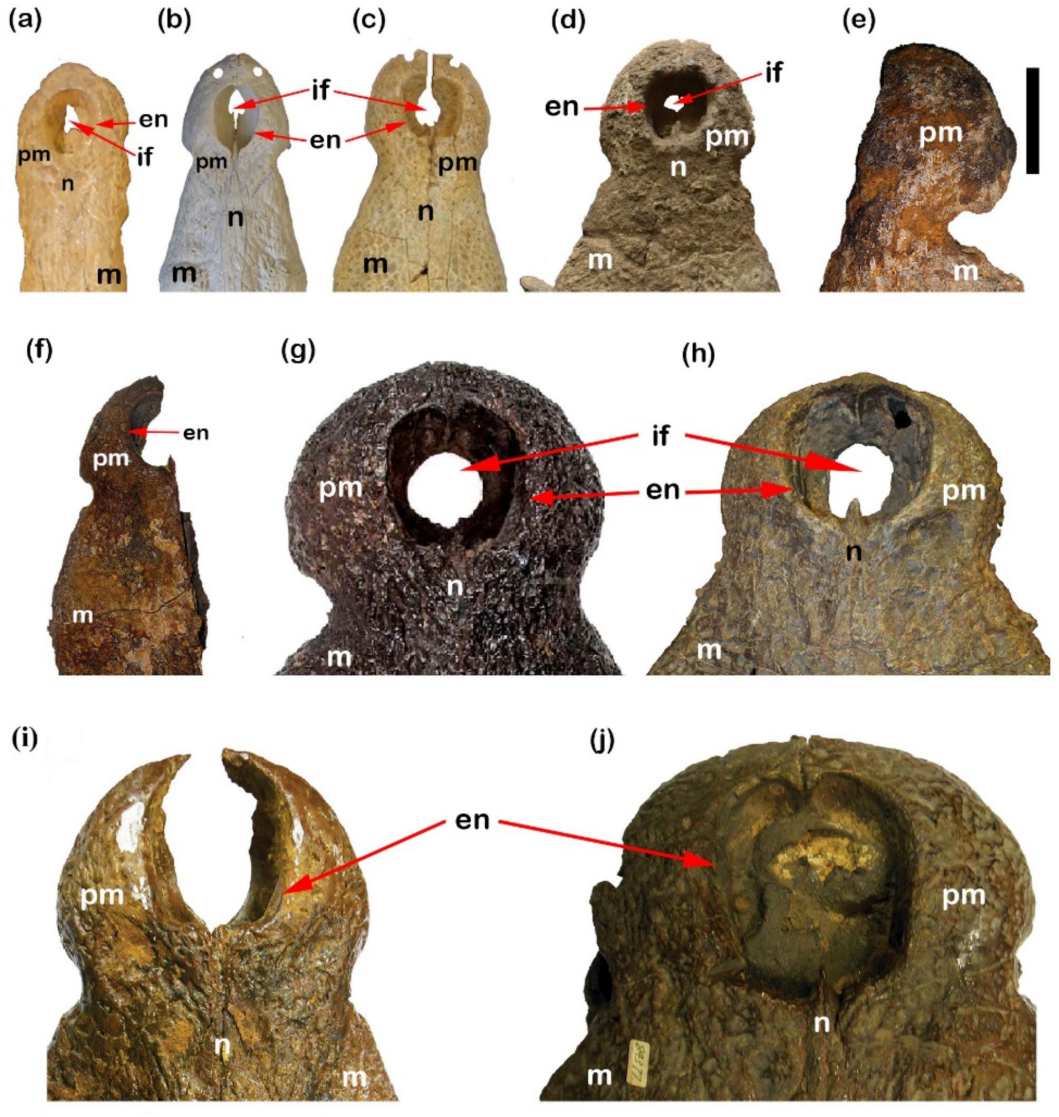
Dorsal crocodylian skulls exhibit differences in premaxilla shape, notch, external naris and the incisive foramen. (**a**) NHMUK R 3322, cast of *Crocodylus articeps*; (**b**) CUVP001, *Crocodylus niloticu*s; (**c**) YPM VP-058532, *Crocodylus megarhinus*; (**d**) CGM84425, *Crocodylus sp*.; (**e**) CGM67110, *Rimasuchus lloydi*; (**f**) CUWM90, *Crocodylus sp.*; (**g**) NHMUK PVR14154, *Rimasuchus lloydi*; (**h**) DPC6610, *Rimasuchus lloydi*; (**i**) CGM73664, *Rimasuchus lloydi*; (**j**) CGM67155, *Rimasuchus lloydi*. Scale bar equals 5 cm.

Eocene *Crocodylus* species from Egypt represented by NHMUK R 3327 (holotype), FARB AMNH 5061, YPM VP-058532 referred to *C. megrahinus* and NHMUK R 3322 referred to *C. articeps* by Andrews^15^, Adams^14^. And there is unidentified specimen from the Egyptian Geological Museum (Egypt) numbered CGM 84425 recorded from the Eocene. Early Miocene specimens^28^ referred to *Rimasuchus lloydi* includes CGM67110, CGM67155, CGM67156, CGM73664, DPC6610, DPC6646, NHMUK PVR14154 and *Crocodylus* specimens include CGM67107, CGM67106, CUWM90 as well as DPC12548^28^. The authors report those morphological difference with the CUVP001 as follows:

Premaxillae in each of CUVP001, the CGM84425, *C. megarhinus*, CUWM90, DPC6610, CGM67110, CGM67155, and in NHMUK PVR14154 are sub-circular and completely round from the anterior end (Fig. 5a–j). But are elongated oval compressed laterally in NHMUK R 3322.

**Fig. 6:**
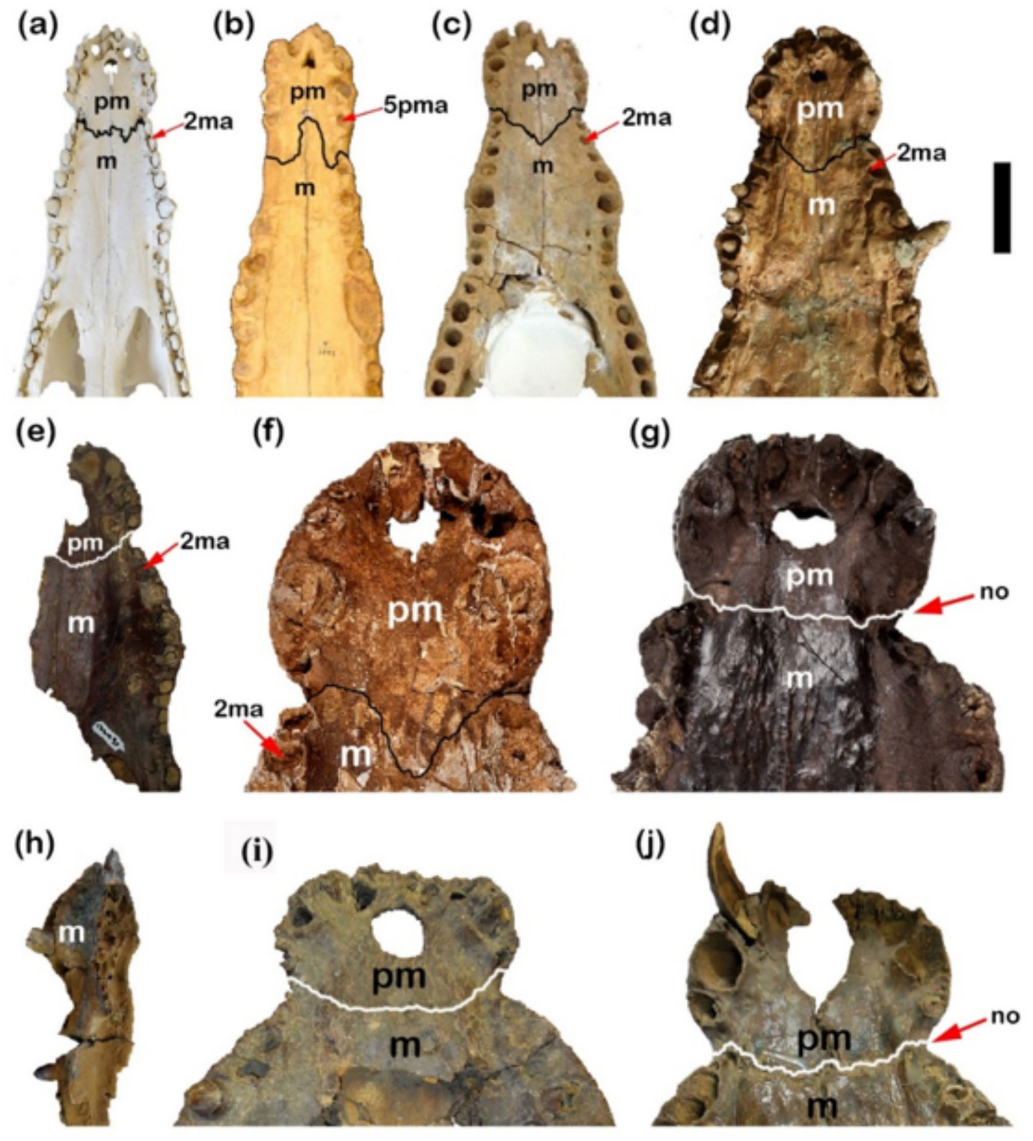
Differences in premaxilla-maxilla suture ventrally, notch between premaxilla and maxilla of Crocodylian skulls. (**a**) CUVP001, *Crocodylus niloticus*; (**b**) NHMUKR 3322, cast of *Crocodylus articeps*; (**c**) YPM VP-058532, *Crocodylus megarhinus*; (**d**) CGM 84425, *Crocodylus sp*.; (**e**) CUWM90, *Crocodylus sp*.; (**f**) NHMUKR. 3327, *Crocodylus megarhinus*; (**g**) NHMUK PVR14154, *Rimasuchus lloydi*; (**h**) DPC12548, *Crocodylus sp*.; (**i**) DPC6610, *Rimasuchus lloydi*; (**j**), CGM73664, *Rimasuchus lloydi*. (no: refers to the notch). Scale bar equals 5 cm.

Also, in the palatal view, the suture between premaxillae and maxillae is an irregular shape in the CUVP001 forming almost a W-shape and it is irregular in CUWM90. While in the NHMUK R 3322, the same suture forms an inverted V-shape. In contrast to case of *C. megarhinus*, and the CGM84425, the posterior branches of the premaxillae act as a barrier to separate the anterior branches of the maxillae which are terminated in the notch between the premaxilla and maxilla as a gentle convex curve oriented posteriorly (V-shape) as shown by Adams^14^. In NHMUK PV R14154, CGM73664 and DPC6610, the suture between the maxilla and premaxilla from ventral view is straight. In CGM67110 and CGM67155, the suture isn’t obvious. In the relation to the extension of premaxilla, from the present study on CUVP001, it was found that it extends at the level of the second maxillary alveolus. This case coincides with the other cases such as CGM84425 and *C. megarhinus*. In contrast, there are other cases of premaxilla terminated. In the ventral view at the level of 5^th^ premaxillary alveolus in the case of NHMUK R 3322. But at the notch between premaxilla and maxilla and sometimes extended until to mid of the 1^st^ maxillary alveolus as in cases of NHMUK PV R14154, CGM73664, DPC6610. While between first and second alveoli in CUWM90 (Fig. 6a–j).

**Fig. 7:**
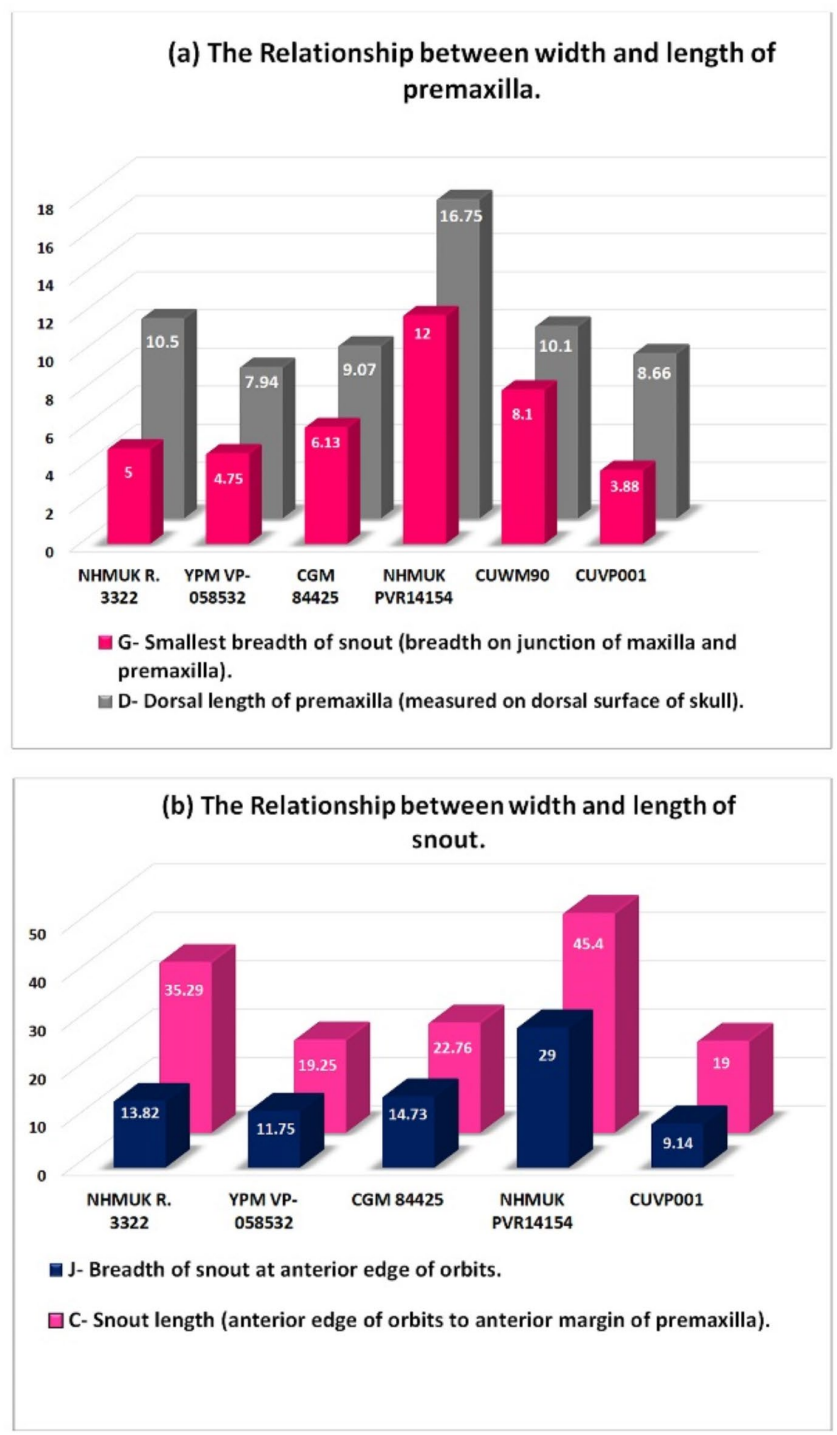
(**a**,**b**) Graphs shows the relationship between width and length of premaxilla and snout.

The premaxillary alveoli are oval and more elongated in CUVP001, while, in NHMUK PVR14154, *C. megharinus*, CGM84425, CUWM90, DPC6610, CGM67110, CGM67155, CGM73664 and NHMUK R 3322 the premaxillary alveoli are circular (Fig. 6a,b,e,g). From the measurements of the premaxillae length, it is found that in CUVP001 the premaxillae length lies between the NHMUK R 3322 and YPM VP-058532 but the width of premaxilla at the level of notch in CUVP001 is smaller than that in YPM VP-058532 and NHMUK R 3322 (Fig. 7a,b).

So, it is indicating that the elongation of the premaxilla in CUVP001 goes beyond that of *C. megarhinus*. But each of the *C. megarhinus* and *C. articeps* premaxilla is broader than that of *C. niloticus*. The width of premaxilla at the notch is closest in both YPM VP-058532 and NHMUK R 3322 but the premaxilla length is longer in the NHMUK R 3322 where was compared to YPM VP-058532. Thus, this confirms that the *C. articeps* premaxilla is more elongated than that of *C. megarhinus*. So, *C. megharinus* and *C. articeps* weren’t synonymous as regarded in^31^. The ratio between the breadths of premaxilla at the level of notch “G” to the dorsal length of premaxilla “D” is 0.4 in both CUVP001 and NHMUK R 3322. While in both YPM VP-058532 and CGM84425 is almost 0.6 and is 0.7 in NHMUK PV R14154 and 0.8 in CUWM90. So, the closest species to CUWM90 is the *R. lloydi*. This ratio shows that *R. lloydi* has the largest breadth “G”. Indeed, *R. lloydi* preserves the proportions of an extreme brevirostrine crocodile as shown by^41^. *C. articeps* and CUVP001 preserves a significant rostral elongation^14^.

**Fig. 8:**
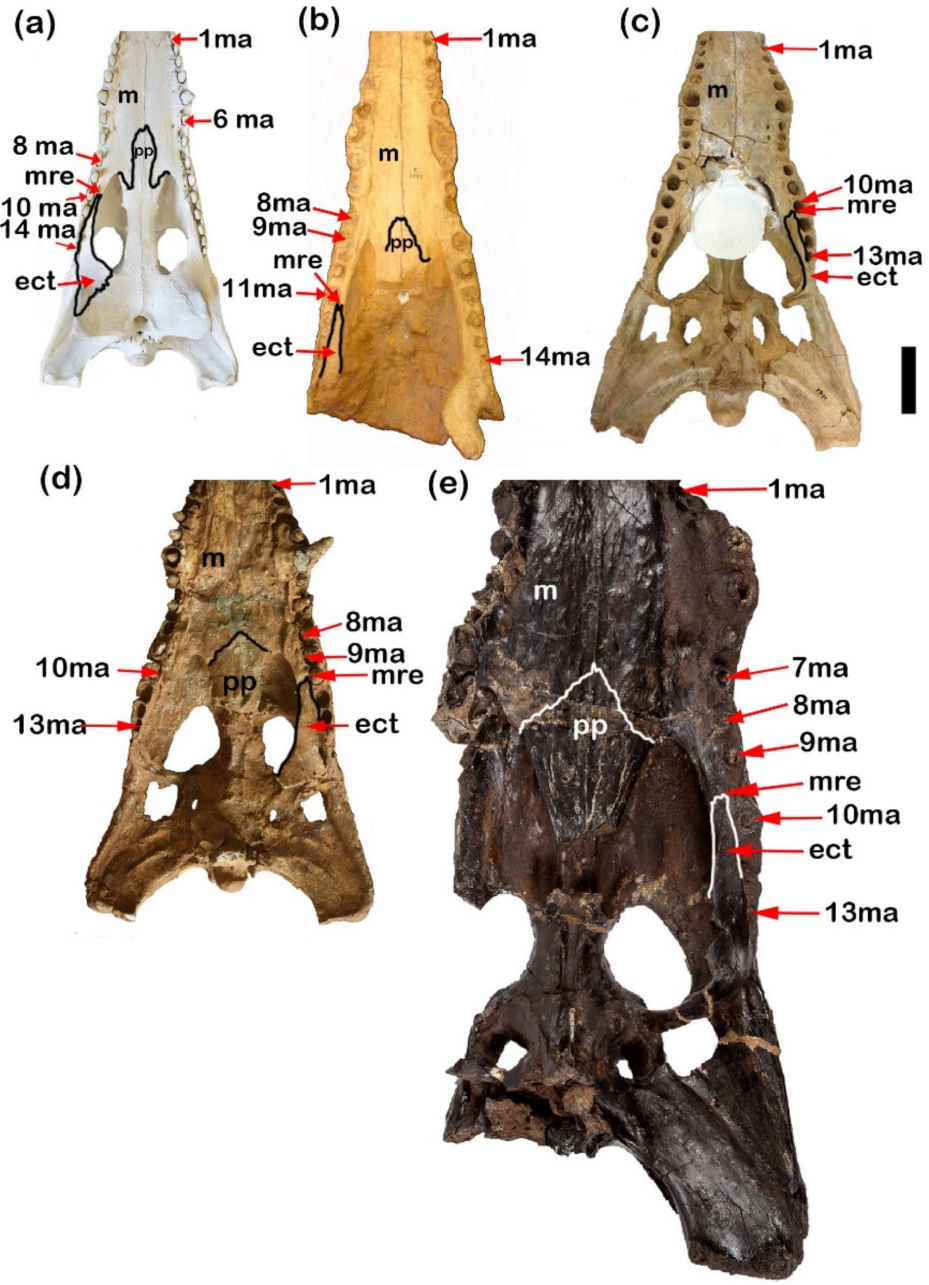
Differences between palatine, maxilla suture, maxillary teeth number, ramus of ectopterygoid and maxilla margins. (**a**) CUVP001, *Crocodylus niloticus*; (**b**) NHMUKR 3322, cast of *Crocodylus articeps*; (**c**) YPMVP-058532, *Crocodylus megarhinus*; (**d**) CGM 84425, *Crocodylus sp*.; (**e**) NHMUKPVR14154, *Rimasuchus lloydi*. (ma. refers to maxillary alveolus). Scale bar equals 5 cm.

The maxillae of CUVP001 contains 14 maxillary alveoli. The same number was found in NHMUK R 3322 and CGM67106. In contrast, CGM84425, FARB AMNH 5061, YPM VP-058532 and NHMUK PV R14154 in which there are 13 maxillary alveoli. This thrown the light onto the possible close relationship between *C. megarhinus* and *Osteolaemus* as suggested by^14^. In the NHMUK R 3327, CGM67107, CUWM90, CGM67123, DPC12548, CGM67110, CGM73664 and DPC6610 the maxillae aren’t complete so, the number of maxillary alveoli doesn’t mentioned. Also, in the CGM67156, DPC6646 and CGM67155 the maxillary alveoli don’t mention due to the maxillae were broken in them. The 5^th^ alveolus is the largest and the fourth one is the second largest in the size in these specimens (Fig. 8a, b), as well as FARB AMNH 5061, CGM67106, CGM67107, CUWM90, DPC12548, CGM73664 and DPC6610. The largest maxillary alveolus can’t be determined in NHMUK R 3327, CGM67123 and CGM67110 due to the maxilla in them isn’t complete.

Laterally the maxillae-palatine suture in the CUVP001 are almost straight, united at the pointed anterior end at midline then posteriorly form a V-shape with suborbital fenestrae. In NHMUK R 3322 the maxillae-palatine suture from anterior and lateral margins is closest to that of a CUVP001 but posteriorly maxillae aren’t complete in NHMUK R332 (Fig. 8a,b). In contrast in FARB AMNH 5061 and NHMUK PV R14154 the palatine-maxilla suture laterally is more irregular and represented in concave and convex curves (Fig. 8a,b,e). In the CGM84425, the maxillae- palatine suture does not appear obviously but is closest to that of *C. megarhinus*. This suture was broken in the YPM VP-058532, CGM67107, CGM67106, CUWM90, CGM67123, DPC12548, CGM67110, CGM73664 and DPC6610. Dorso-laterally, the maxilla margins show the broadest curves in case of NHMUK PV R14154, DPC12548, then the maxilla margins followed by CUWM90, *C. megarhinus* and CGM84425. In contrast to the CUVP001 and NHMUK R 3322 in which curved narrowly (Figs. 6e,h and 8a–e). The maxilla anterior margins in the CGM67107 and CGM67106 aren’t obvious and are broken in the CGM67123, CGM67110, CGM73664 and DPC6610. In CGM67110, DPC6610, CGM73664, CUWM90, CGM67155 and NHMUK PV R14154 the occlusal groove (notch between the premaxilla and the maxilla) is deeper than CUVP001, CGM84425 and *C. megarhinus* and the less deep appears in NHMUK R 3322 (Figs. 5e,k and 6a–g,j,k). This notch is broken in the CGM67107, CGM67106, CGM67123, DPC12548, CGM67156 and DPC6646. It is found that CGM67107 is identical to the description of the NHMUK PV R14154 from 4^th^ to 10^th^ maxillary alveoli, the maxillae of both get narrow at the level of the 7^th^ maxillary alveoli, while the 9^th^ and 10^th^ maxillary alveoli are larger than the 7^th^ and 8^th^ maxillary alveoli. In addition to that the 5^th^ maxillary alveolus is the largest one. The alveoli of the both are circular in the shape. So, CGM67107 refers to *R. lloydi*. Both of CGM67106 and CUWM90 are identical through the close alveoli from each other in between the 6^th^ to 9^th^ alveoli in contrast to that of NHMUK PV R14154. But the shape of the alveoli is the same. Also, both of CGM67123 and CGM67106 have the same morphological feature of the posterior part which represented in the shape of teeth and alveoli which are more elongated and these features is similar to CUVP001. These two previous cases are contrast to *R. lloydi*. Both of the CGM67106, CGM67123 as well as CUWM90 are probably referred to new species which contain 14 alveoli from Miocene epoch and preserves the broadest snout. This snout is characteristic of the Miocene crocodilian *R. lloydi*. The measurements revealed that although the *C. articeps* snout breadth “J” lies between YPM VP-058532 and CGM84425, the snout length “C’” of the *C. articeps* is longer than and YPM VP-058532 and CGM84425 (Fig. 7b). That graph shows that the snout length increases parallel with the snout width in the specimens except in the *C. articeps* which is more elongated. The present study on CUVP001 and YPM VP-058532 have the closest snout length, but, CUVP001 showed the less breadth than the YPM VP-058532. The width across 5^th^ maxillary teeth “canines” in CUVP001, CGM84425, NHMUK R 3322, CUWM90 and *C. megarhinus* are closer and does not exceed the range of NHMUK PV R14154. Through the ratio between smallest breadth of snout “G”/breadth of snout at fifth maxillary tooth “H” it is found that NHMUK PV R14154 has the ratio 0.4 and CUWM90 has the ratio 0.71 while CGM84425 and CUVP001 have the same ratio 0.5. But YPM VP-058532, NHMUK R 3322 have the approximately ratio 0.6. This ratio refers that the CUVP001is closer to CGM84425 than YPM VP-058532 and NHMUK R 3322. While the ratio of the NHMUK PV R14154 is the smallest one due to it contains the largest breadth of snout at 5^th^ maxillary alveoli.

The suture through the medial margin of the nasal is obvious in CUVP001, *C. megarhinus*, CGM73664, CGM67155 and CGM84425. The reverse condition is found in cases of NHMUK R 3322 and NHMUK PV R14154 which the suture is obliterated along part of the shared medial margin of the nasal. The nasals of CUVP001 and NHMUK R 3322 reach their greatest width at the level of the 9^th^ maxillary alveolus at the level of the most anterior margin of the prefrontal. On the other hand, the nasal of NHMUK PVR14154 reaches to its greatest width almost anteriorly far away from the level of the beginning of the prefrontal. While in YPM VP-058532, the greatest width extended at the level of the most anterior margin of lacrimals. The other condition was found in NHMUK R 3327 and FARB AMNH 5061 where their nasal extension reach the greatest width at the level of 4^th^ maxillary alveolus. In the CGM84425 is not clear. Nasal is broken in all other specimens.

Lacrimal has the same morphological feature in CUVP001, YPM VP-058532, FARB AMNH 5061, CGM84425 and NHMUK PVR14154. It isn’t obvious in NHMUK R. 3327, CGM67106, CGM73664, NHMUK R 3322, and CUWM90 and broken in the others.

**Fig. 9:**
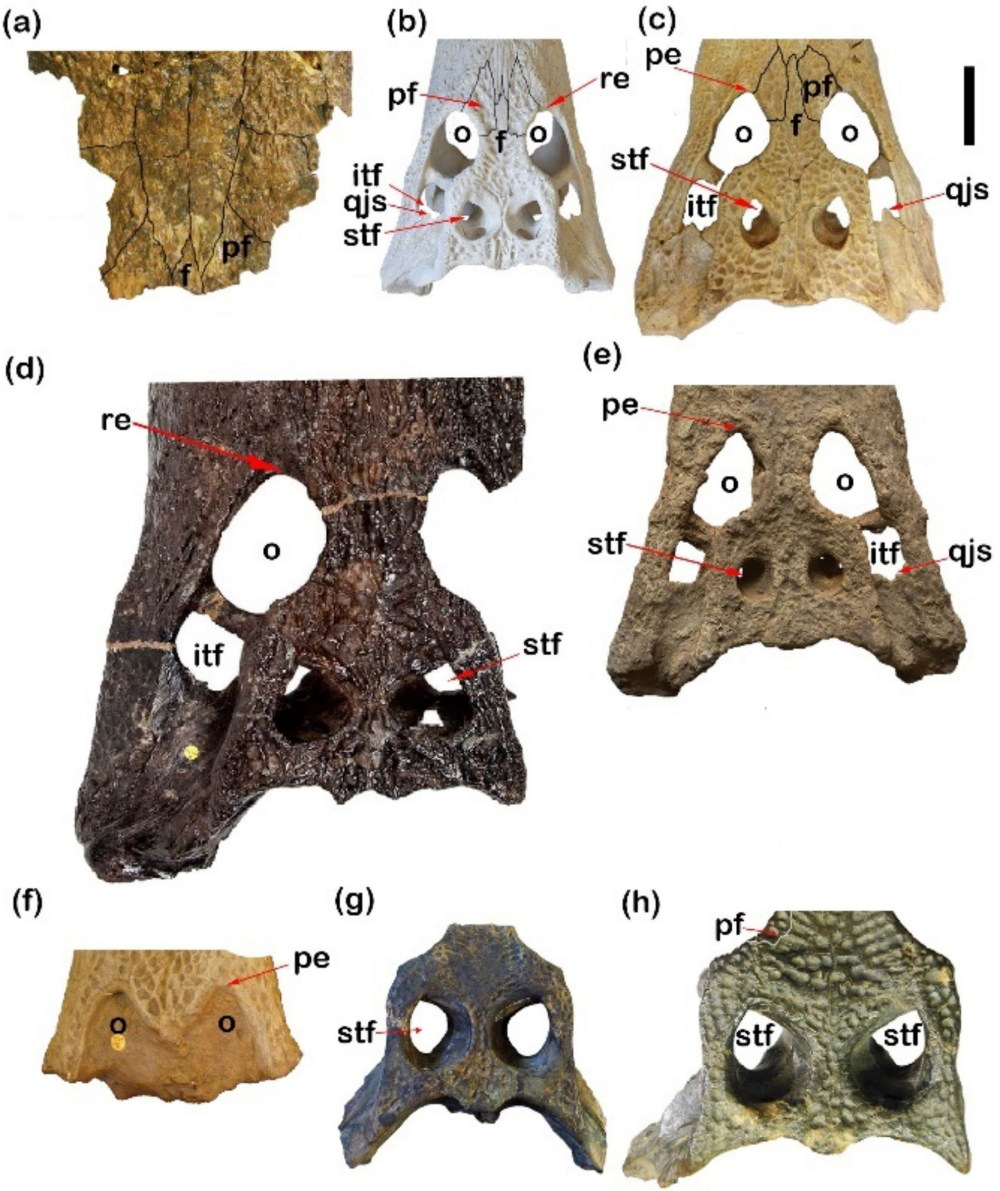
Differences in orbit, frontal shape, space between supratemporal fenestrae and quadratojugal spine of Crocodylian skulls. (**a**) CGM73664, *Rimasuchus lloydi*; (**b**) CUVP001, *Crocodylus niloticus*; (**c**) YPM VP-058532, *Crocodylus megarhinus*; (**d**) NHMUK PVR14154, *Rimasuchus lloydi*; (**e**) CGM 84425, *Crocodylus* sp.; (**f**) NHMUK R 3322, cast of *Crocodylus articeps*; (**g**) DPC6646, *Rimasuchus lloydi*; (**h**) CGM67156, *Rimasuchus lloydi*. (qjs, quadratojugal spine; pe. refers to the pointed end and re. refers to rounded end). Scale bar equals 5 cm.

Prefrontal has the same shape in CUVP001, CGM73664, FARB AMNH 5061 and YPM VP-058532. In CUVP001 and CGM73664 the anterior end of the prefrontal lies above the frontal, but the anterior end of the prefrontal and frontal lies at the same level in NHMUK R 3327, YPM VP-058532 and FARB AMNH 5061 (Fig. 9b,c). It isn’t obvious in CGM67156, NHMUK R 3327, NHMUK R 3322, CUWM90, CGM67156, CGM84425 and NHMUK PVR14154, and broken in the rest specimens.

Jugal has the same morphological feature in CUVP001, YPM VP-058532, FARB AMNH 5061, CGM84425 and NHMUK PVR14154. It isn’t complete in CGM67106, CUWM90, CGM67123 and NHMUK R 3322, and broken in the other specimens.

In the quadratojugal of CUVP001, there is a spine that enters infratemporal fenestra on each side. On contrary in the case CGM84425, this is not obvious on both two sides. It appears somewhat on the left side and is absent on the right one. The same case showed from the YPM VP-058532. Adams^14^ demonstrated that in the juvenile of *C. megarhinus* the quadratojugal spine is prominent but absent and is not obvious in the adult *C. megarhinus*. In the NHMUK R 3322, the CGM67106, CGM67107, CGM67123, DPC12548, CGM67110, DPC6610, CGM73664, CGM67155, NHMUK R 3327 and CUWM90 the quadratojugal is broken. In FARB AMNH 5061, CGM67156, DPC6646 and NHMUK PVR14154 a quadratojugal spine is not clear (Fig. 9a–e). The *C. niloticus* compared with *C. megarhinus* and CGM84425 shows that the width of the skull across the anterior end of orbits in relation to the width of the skull across the quadratojugals is the same 0.6 as where Mook^16^ suggested that *C. megarhinus* is closely related to *C. niloticus*, while it is wider within *R. lloydi* which equal to 0.7.

Quadrate has the same morphological feature in CUVP001, YPM VP-058532, FARB AMNH 5061, CGM67156, DPC6646, CGM84425 and NHMUK PVR14154 and damaged in other specimens.

Postorbital has the same morphological feature in CUVP001, YPM VP-058532, FARB AMNH 5061, CGM67156, DPC6646, CGM84425 and NHMUK PVR14154, and damaged in other specimens.

The palatine is a long bone due to its anterior long process in case of CUVP001. In case of NHMUK R 3322, FARB AMNH 5061, and CGM84425 and NHMUK PV R14154 the palatine is short due to its short process. The palatine-maxilla suture intersects with the suborbital fenestrae at the beginning of the 9^th^ maxillary alveolus in CUVP001. But this suture was found at the middle of the 9^th^ maxillary alveolus in FARB AMNH 5061, CGM84425 and between 8^th^ and 9^th^ maxillary alveoli in NHMUK PV R14154. Lastly, it was not clear in NHMUK R 3322. In CUVP001, the anterior process merges at an acute tip at the level of the 6^th^ maxillary alveolus approximately above the anterior-most tip of the suborbital fenestrae which is at the beginning of the 9^th^ maxillary alveolus. Also, anterior process in each of NHMUK R 3322, FARB AMNH 5061 and CGM84425 merges at the level of the 8^th^ maxillary alveolus approximately above the anterior tip of the suborbital fenestrae which located at the 9^th^ maxillary alveolus. In NHMUK PV R14154 the anterior process of palatine merges at an acute tip between the 6^th^ and 7^th^ maxillary alveoli approximately above the anterior-most tip of the suborbital fenestrae which is between the 8^th^ and 9^th^ maxillary alveoli (Fig. 8a–e). So, the anterior process of palatine in CUVP001 is long. Palatine is broken in other specimens.

Ectopterygoids extended anteriorly to the border of the 10^th^ maxillary alveolus in case of CUVP001 and FARB AMNH 5061, CGM84425, while in NHMUK R 3322 this bone extended to the border of the 11^th^ maxillary alveolus. In NHMUK PV R14154 extended anteriorly to the border between the 9^th^ and 10^th^ maxillary alveoli. In the present study on CUVP001 and CGM67123 the maxillary ramus of the ectopterygoid lies parallel to the last five maxillary alveoli and has a forked anterior tip. In other cases, it lies parallel to the last four maxillary alveoli such as FARB AMNH 5061, CGM84425, CGM67106 and NHMUK R 3322. A forked anterior tip appears in FARB AMNH5061, CGM84425 which referred to *C. megarhinus*. But that finding conflicts with Adams^14^ who demonstrated that *C. megarhinus* doesn’t contain a forked ectopterygoid. Also, Brochu^31^, showed that this forked anterior tip of ectopterygoid is a specific character of the genus *Crocodylus*. While the maxillary ramus of the left ectopterygoid lies parallel to the last four maxillary alveoli and bears an unforked anterior tip in NHMUK PV R14154 (Fig. 8a–e). Ectopterygoid is damaged in others.

Petrygoid has the same morphological feature in CUVP001and FARB AMNH 5061 and it is broken in the other describe materials herein.

Vomer has the same morphological feature in CUVP001 and FARB AMNH 5061. It is broken in other described materials herein.

The supraoccipital has the same shape from dorsal view in CUVP001, FARB AMNH 5061, DPC6646, CGM84425, YPM VP-058532 and CGM67156. The supraoccipital has a heart shape in CUVP001, NHMUK PVR14154 which is less length than that in CGM84425, YPM VP-058532 and CGM67156. In CUVP001 the parietal-squamosal suture exists interiorly related to lateral suture of the supraoccipital. On contrary the suture of supraoccipital in NHMUK PVR14154 and CGM67156 is continuous with parietal-squamosal suture from dorsal view. It is not obvious in YPM VP-058532 and CGM84425. The supraoccipital is broken in the NHMUK R 3322 DPC6610, CGM67106, CGM67107, CGM67123, DPC12548, CGM67110, CGM73664, CGM67155, NHMUK R 3327, CUWM90 and part of the triangular wedge of NHMUK PVR14154. The triangular wedge of the supraoccipital from dorsal view in CUVP001 is less wide than that in YPM VP-058532, CGM84425, DPC6646 and CGM67156. In addition to the YPM VP-058532 showed the widest triangular wedge of supraoccipital. By the comparison of the width of triangular wedge of the supraoccipital from dorsal view to the width of the bar between supratemporal fenestrae between the different specimens it is found that, the wedge width is larger than the bar width CUVP001, YPM VP-058532 and DPC6646. On the other hand, the bar width is larger than the wedge width in CGM84425 and CGM67156.

Exooccipital has the same shape in CUVP001, NHMUK PVR14154, DPC6646, CGM84425, YPM VP-058532 and CGM67156. It is not clear in the FARB AMNH 5061. It is damaged in others.

Basioccipital is similar in the morphology in the CUVP001, DPC6646, CGM84425, YPM VP-058532 and CGM67156. It is not clear and complete in the NHMUK PVR14154 and FARB AMNH 5061. It is damaged in others.

Prootic is similar in the morphology in the CUVP001, DPC6646, NHMUK PVR14154, CGM84425, YPM VP-058532 and CGM67156. It is not clear FARB AMNH 5061. It is broken in others.

Laterosphenoid is similar in the morphology in the CUVP001, DPC6646, NHMUK PVR14154, CGM84425, YPM VP-058532 and CGM67156. It is not clear FARB AMNH 5061. It is broken in the other described materials herein.

Basisphenoid is similar in the morphology in the CUVP001, DPC6646, CGM84425, YPM VP-058532 and CGM67156. It is not clear FARB AMNH 5061 and it is not complete in NHMUK PVR14154. It is broken in the others.

The frontal has the same shape in CUVP001, YPM VP-058532 and FARB AMNH5061. In CUVP001 and CGM73664 the anterior end of the frontal lies below the prefrontal but the anterior end of the frontal and prefrontal lies at the same level in NHMUK R 3327, YPM VP-058532 and FARB AMNH 5061 (Fig. 9b,c). In the same time the anterior end of the frontal is not clear in NHMUK R 3322, CGM84425 and NHMUK PVR14154 and it isn’t complete in DPC6646 and CGM67156 and broken in other described materials.

Parietal is similar in the morphology in the CUVP001, DPC6646, CGM84425, YPM VP-058532, CGM67156, FARB AMNH 5061 and NHMUK PVR14154. It is broken in the others.

Squamosal is similar in the morphology in the CUVP001, DPC6646, CGM84425, YPM VP-058532, CGM67156, FARB AMNH 5061 and NHMUK PVR14154. It is broken in others.

There are differences in the shape and size of the external naris between CUVP001, DPC6610, CGM73664, CUWM90, CGM67155, and *C. megarhinus*, NHMUK R 3322, CGM84425 and NHMUK PVR14154 (Fig. 5a–d,g,h,j).

**Fig. 10:**
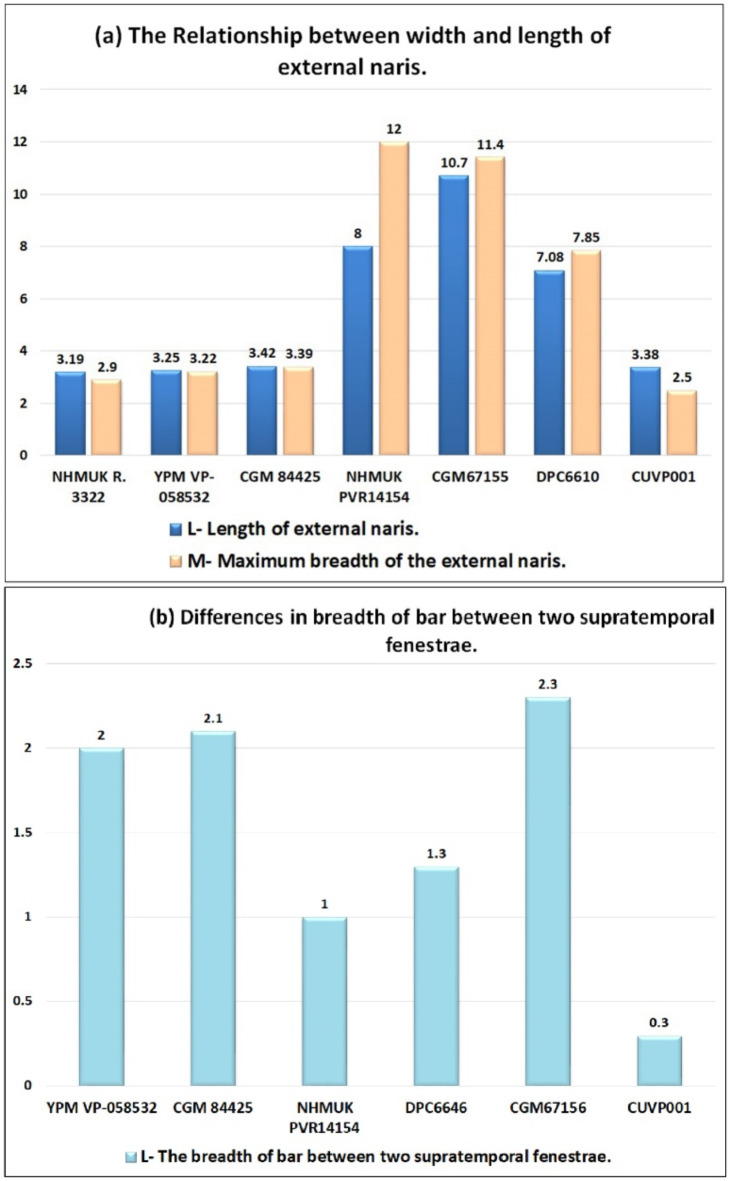
(**a**) Graph shows the relationship between the width and length of the external naris. (**b**) Graph shows differences in the breadth of the space between the 2 fenestrae.

The CUVP001 and CGM73664 have a pear-shaped and are bisected posteriorly by anterior projections of the nasals. In *C. megarhinus*, CGM84425, CGM67155, DPC6610, CUWM90 and NHMUK PVR14154, the external naris is bisected posteriorly by anterior projections of the nasals, and, the overall shape resembles an apple. In the NHMUK R 3322, it is found that the shape of the external naris looks like that of the CUVP001 but, when compared it there is no naris bisected posteriorly by anterior projections of the nasals. It is found that the length of external naris in CUVP001 is larger than their width but vice versa in case as the NHMUK PVR14154, CGM67155 and DPC6610. In other cases, such as CGM84425, YPM VP-058532 and the NHMUK R 3322 the width of the external naris is equal in length (Fig. 10a,b). It is broken in the other described materials.

Foramen for the first dentary tooth isn’t clear in the fossils.

The incisive foramina in the CUVP001, and *C. megarhinus* have three posterior lobes (Fig. 5a–d,g,h). In the NHMUK R 3322 only it has two posterior lobes. But in each of the CGM84425, DPC6610, NHMUK PVR14154 the formina are semi-circular in shape. It is not clear in CGM67155. It is broken in the rest specimens.

The anterior side of the orbit in CUVP001 and NHMUK PVR14154 is round. In the NHMUK R 3322, only the anterior margin of the orbit is preserved with rounded borders and slightly pointed anterior ends. Orbits in the CGM84425, YPM VP-058532 and FARB AMNH 5061 referring as in Adam^14^ are oval in shape with rounded borders and slightly pointed anterior ends (Fig. 9a–e). It is damaged in the rest materials.

The shape of the supratemporal fenestrae is the same in CUVP001, *C. megarhinus*, CGM67156, DPC6646, CGM84425 and NHMUK PVR14154. In the NHMUK R 3322 the supratemporal fenestrae is damaged. The space between the two fenestrae is very narrow in *C. niloticus*, DPC6646 and *Rimansuchus lloydi* while it is wide in CGM67156, *C. megarhinus* and CGM84425 (Figs. 9a–g and 10b). It is broken in the others.

Infratemporal fenestrae are similar in the morphology in the CUVP001, CGM84425, YPM VP-058532, FARB AMNH 5061 and NHMUK PVR14154. It is broken in the others.

The external otic aperture in each of CGM84425, *C. megarhinus*, CGM67156 and in NHMUK PVR14154 is identical to that of the CUVP001. It is not clear in DPC6646. It is damaged in the rest specimens.

Olfactory opening is similar in the morphology in the CUVP001, DPC6646, CGM67156, and YPM VP-058532. It is not clear in CGM84425, FARB AMNH 5061 and NHMUK PVR14154. It is damaged in the rest specimens.

Optic foramen is similar in the external feature in the CUVP001, DPC6646, CGM67156, and YPM VP-058532. It is not clear in CGM84425, FARB AMNH 5061 and NHMUK PVR14154. It is damaged in the other specimens.

Orbitotemporal foramen is similar in the external feature in the CUVP001, and YPM VP-058532. It is not clear in DPC6646, CGM67156, CGM84425, FARB AMNH 5061 and NHMUK PVR14154. It is damaged in the other specimens.

Post temporal fenestrae is similar in the morphology of the CUVP001, CGM67156, NHMUK PVR14154, and YPM VP-058532. It is not clear in DPC6646, CGM84425 and FARB AMNH 5061. It is damaged in the other desrcibed specimens.

Palatal fenestrae have the same shape in the CUVP001, FARB AMNH 5061, CGM84425 and NHMUK PVR14154. It is not complete in CUWM90, NHMUK R. 3322, NHMUK PVR14154, CGM84425 and CGM67106. It is damaged in the rest specimens.

Trigeminal foramen has the same shape in the CUVP001, CGM84425, CGM67156 and YPM VP-058532. It is not clear in NHMUK PVR14154, FARB AMNH 5061and DPC6646. It is damaged in the rest specimens.

The shape of choana in the CUVP001 looks like the heart where the posterior border of it is concave toward the exterior. But, in the FARB AMNH 5061 the shape of choana is triangular in the shape and its posterior border is straight where Adams^14^ confirmed that the choana does not appear to be notched along the posterior rim. The choana is damaged in the other described specimens herein.

Median and lateral Eustachian canals have the same shape in the CUVP001, CGM84425, FARB AMNH 5061, DPC6646 and YPM VP-058532. It is not clear in CGM67156. It is damaged in the rest specimens.

Foramen aerium has the same shape in the CUVP001 and NHMUK PVR14154. It is not clear in CGM84425, FARB AMNH 5061, DPC6646 and YPM VP-058532. It is damaged in the rest specimens.

Foramen magnum is sub-circular in CUVP001 while it has anterior concave curve and posterior V-shape in CGM67156 and YPM VP-058532. In CGM67156 and YPM VP-058532, the width of the foramen magnum is larger than their length. In the CUVP001, the width of the foramen is almost equal to its length. So, the CUVP001 has almost the same width of the foramen magnum in case of YPM VP-058532 but the foramen magnum length in CUVP001 is longer than that in YPM VP-058532. While CGM67156 foramen magnum has approximately the twice width of that in CUVP001, so foramen magnum of CGM67156 is broader than that in CUVP001. The distance of the exoccipital separate between supraoccipital and foramen magnum is larger in the CUVP001 than in the CGM67156 and YPM VP-058532. It is not clear in DPC6646, CGM84425 and FARB AMNH 5061 and not complete in NHMUK PVR14154. It is damaged in the rest specimens. Occipital condyle has the same shape in the CUVP001, CGM84425, FARB AMNH 5061, CGM67156 and YPM VP-058532. It is not complete in DPC6646 and NHMUK PVR14154. It is damaged in the rest specimens. Its function is articulation with the first vertebrae so, it is found that it increases in size as the skull size increases.

**Table 1:** Cranial measurements of (*Crocodylus niloticus*) from lake Nasser, measurements illustrated in (Fig. 11) and specimens included in this study. All measurements in cm; missing measurements indicated by “–”.

Specimen number	NHMUK *R* 3322	YPM VP-058532 *C. megarhinus* ^(14)^	CGM 84425	FARB AMNH 5061 ^(28)^	NHMUK PVR14154 *R*. *lloydi*	CGM67155 *R*. *lloydi* ^(28)^	DPC6610 *R*. *lloydi* ^(28)^	DPC6646 *R*. *lloydi* ^(28)^	CGM67156 Indeterminate genus ^(28)^	CUWM90 Indeterminate genus ^(28)^	CUVP001 *C. niloticu* *s*
A-Head length (anterior border premaxilla to posterior margin supraoccipital)	–	34	37.63	–	69.5	–	–	–	–	–	29.7
B-Greatest lateral length of skull (anterior margin of premaxilla to posterior margin of quadrate)	–	35.87	40.39	–	74.45	–	–	–	–	–	31.4
C-Snout length (anterior edge of orbits to anterior margin of premaxilla)	35.29	19.25	22.76	–	45.4	–	–	–	–	–	19
D-Dorsal length of premaxilla (measured on dorsal surface of skull)	10.5	7.94	9.07	–	16.75	–	–	–	–	10.1	8.66
E-Length of external naris	3.19	3.25	3.42	–	7.45	10.7	7.08	–	–	–	3.38
F-Maximum breadth of the external naris	2.9	3.22	3.39	–	8	11.4	7.85	–	–	–	2.5
G-Smallest breadth of snout (breadth on junction of maxilla and premaxilla)	5	4.75	6.13	–	12	–	–	–	–	8.1	3.88
H-Breadth of snout at fifth maxillary tooth	8.2	7.97	10.92	–	26	–	–	–	–	11.3	6.77
J-Breadth of snout at anterior edge of orbits	13.82	11.75	14.73	–	29	–	–	–	–	–	9.14
K-Breadth at anterior edge of cranial roof (at postorbitals)	–	9	8.94	–	14.4	–	–	10.674	15.598	–	6.57
L-The breadth of bar between two supratemporal fenestrae	–	2	2.1	2.4	1	–	–	1.3	2.3	–	0.3
M-Greatest breadth at posterior edge of cranial roof (at squamosal protuberances)	–	10.25	10.52	–	17.95	–	–	12.022	16.713	–	7.1
N-Greatest posterior breadth of skull at quadratojugals	–	18.225	22.63	–	41	–	–	–	–	–	14.14
O-The width of the triangular wedge of supraoccipital from dorsal view	–	2.75	1.5	–	–	–	–	1.7	1.6	–	0.77
P-The angle made by the intersection of a line parallel to the skull table lateral margin and sagittal plane	–	9°	9°	–	12°	–	–	–	–	–	7°
Q-The supraoccipital length from occipital view	–	1.5	–	–	–	–	–	–	2.08	–	1.15
R-Width of the foramen magnum	–	1.87	–	–	–	–	–	–	3.1	–	1.7
S-Length of the foramen magnum	–	0.87	–	–	–	–	–	–	1.7	–	1.4
T-The distance of the exoccipital separate between supraoccipital and foramen magnum	–	0.46	–	–	–	–	–	–	0.5	–	1.2

**Fig. 11:**
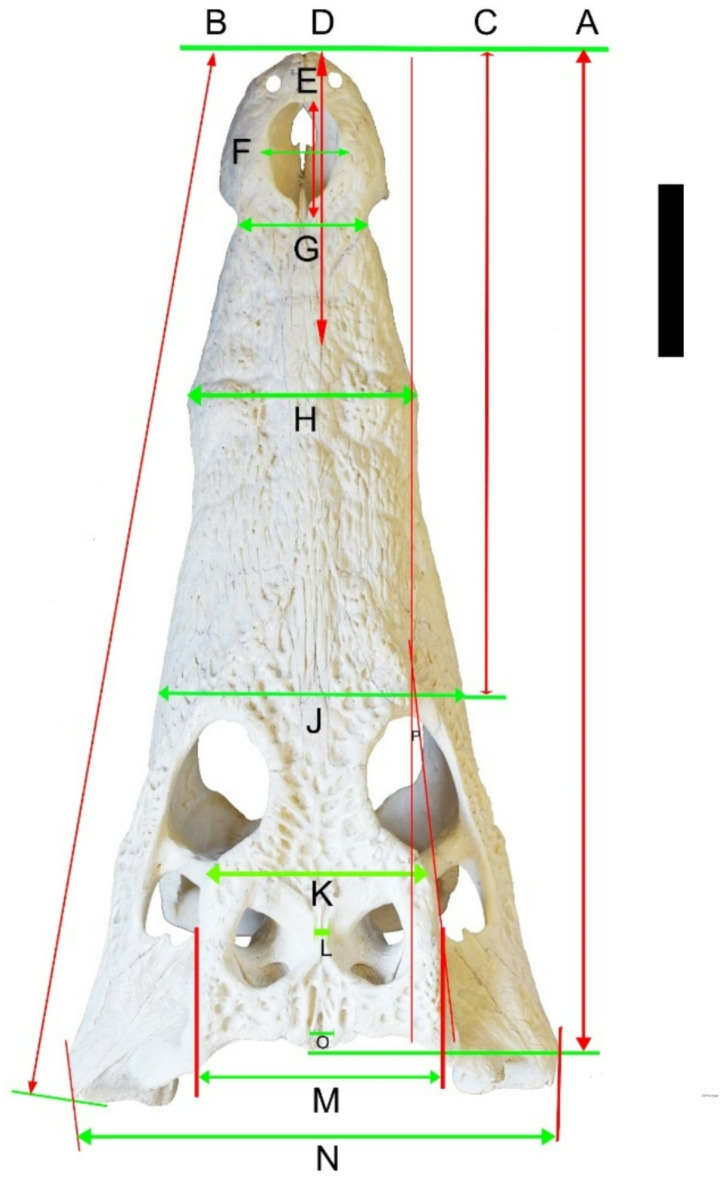
Cranial measurements of *Crocodylus niloticus* skull from dorsal view, each letter illustrated in (Table 1). Scale bar equals 5 cm.

The angle made by the intersection of a line parallel to the skull table lateral margin and sagittal plane is larger in NHMUK PV R14154, CGM84425, YPM VP-058532 than in CUVP001. This angle in CGM84425 equals 9° to that of YPM VP-058532 see (Table 1). From morphological features and different ratios of measurements, it is found that CGM 84425, *Crocodylus* sp. from Eocene epoch refers to *C. megarhinus*.

From studying the morphological features of the lower jaw of the Eocene specimens which included NHMUK R 3324, NHMUK R 3323 cast of C. 10065, NHMUK R 3105 and CGM (C. 10065) are represented *C. articeps*, NHMUK R 3328 and FARB AMNH 5095 are represented *C. megarhinus*, NHMUK R 3104 is represented *Crocodylus* species. In addition to the lower jaw of the Miocene specimens which included CGM67117, CGM67118, and CGM67155 which are represented *Rimasuchus lloydi*.

The comparison between the previous specimens and CUVP001 demonstrated the following:

The complete mandible of CUVP001 are identical to *C. articeps*, NHMUK R 3328 and FARB AMNH 5095 in the number of the alveoli. All contain 15 alveoli. The alveoli shape are elongated and more oval in NHMUK R 3104, CUVP001, and *C. articeps*. In contrast to their shape in the NHMUK R. 3328, FARB AMNH 5095, CGM67117, CGM67118, and CGM67155 which are circular. In *C. niloticus; C. articeps; C. megarhinus; R. lloydi* and *Crocodylus* sp. lower jaws, the 4^th^ alveolus is the largest one. The smallest alveolus is the third in CUVP001, NHMUK R 3328, FARB AMNH 5095, NHMUK R 3324, NHMUK R 3323, NHMUK R. 3105 and CGM (C. 10065). In addition to that, there are differences in the second large alveolus after the 4^th^ one. The 1^st^ alveolus is the second large one in CUVP001 and *C. articeps*. In contrast to the previous case in NHMUK R 3328 and FARB AMNH 5095 which is 10^th^ alveolus. The third and fourth alveoli are very close to each other. They were only separated by a bony septum not the same in all specimens. The bony septum with a width of 0.3 cm in CUVP001 and ranging from 0.4 to 0.6 cm in *C. articeps*, 0.5 in CGM67117 and 0.6 cm is shown in NHMUK R 3328. The shape of the external mandibular fenestrae is not the same in all specimens. In CUVP001, the external mandibular fenestrae is identical to that of NHMUK R 3328 and FARB AMNH 5095^14^ which have a broad rounded posterior margin and narrow pointed anterior edge. In the case of *C. articeps* and NHMUK R 3104 this fenestra looks like a convex lens which has narrow pointed anterior and posterior margins then broad at the middle of the fenestrae.

The suture between the suraangular and angular is straight. It intersects the external mandibular fenestrae in the middle of its posterior margin in CUVP001, NHMUK R 3328 and FARB AMNH 5095. In contrast, the suture is straight and then turned upward making a concave curve in *C. articeps* and NHMUK R 3104. From the dorsal view of mandible of NHMUK R 3328 the symphysis length is 14.28 cm and the width of each lower jaw at the posterior of symphysis equal 7 cm. From the dorsal view of mandible of NHMUK R 3324, NHMUK R 3323, NHMUK R 3105 the symphysis length are 11.5 cm, 8.4 cm and 12.8 cm respectively and the width of each lower jaw at the posterior of symphysis of these specimens equal 4 cm, 3.8 cm and 5.3 cm respectively. The lower jaw is thick with a symphysis long measures 4 cm, 7.5 cm and 16.4 cm respectively in CGM67117, CGM67118 and CGM67155. The symphysis width measures 5.1 cm in CGM67118. While in CUVP001 is narrow with a short symphysis measured 3.2 cm in length and 1.6 cm in width. So, the least symphysis length is in CUVP001. The anterior part of mandible is very wide in *C. articeps*, CGM67118 and NHMUK R 3328 but is narrow in CUVP001. In CUVP001, the fourth mandibular tooth is sharp and less wide and bulbous than of the CGM67155. The fourth mandibular tooth measured 0.8 cm in width in the CUVP001 while in CGM67155 it attained 1.8 cm in diameter. It is found that the shape of preserved teeth is conical in NHMUK R 3324, NHMUK R 3323 and CGM (C. 10065). They are not obvious in NHMUK R 3104 and FARB AMNH 5095. None of the teeth are existed in NHMUK R. 3328 and NHMUK R. 3105. So, the mandibular teeth are wide and thick in *C. articeps*, in contrast to the CUVP001 which are thin and more concavely curved toward the interior side.

**Fig. 12:**
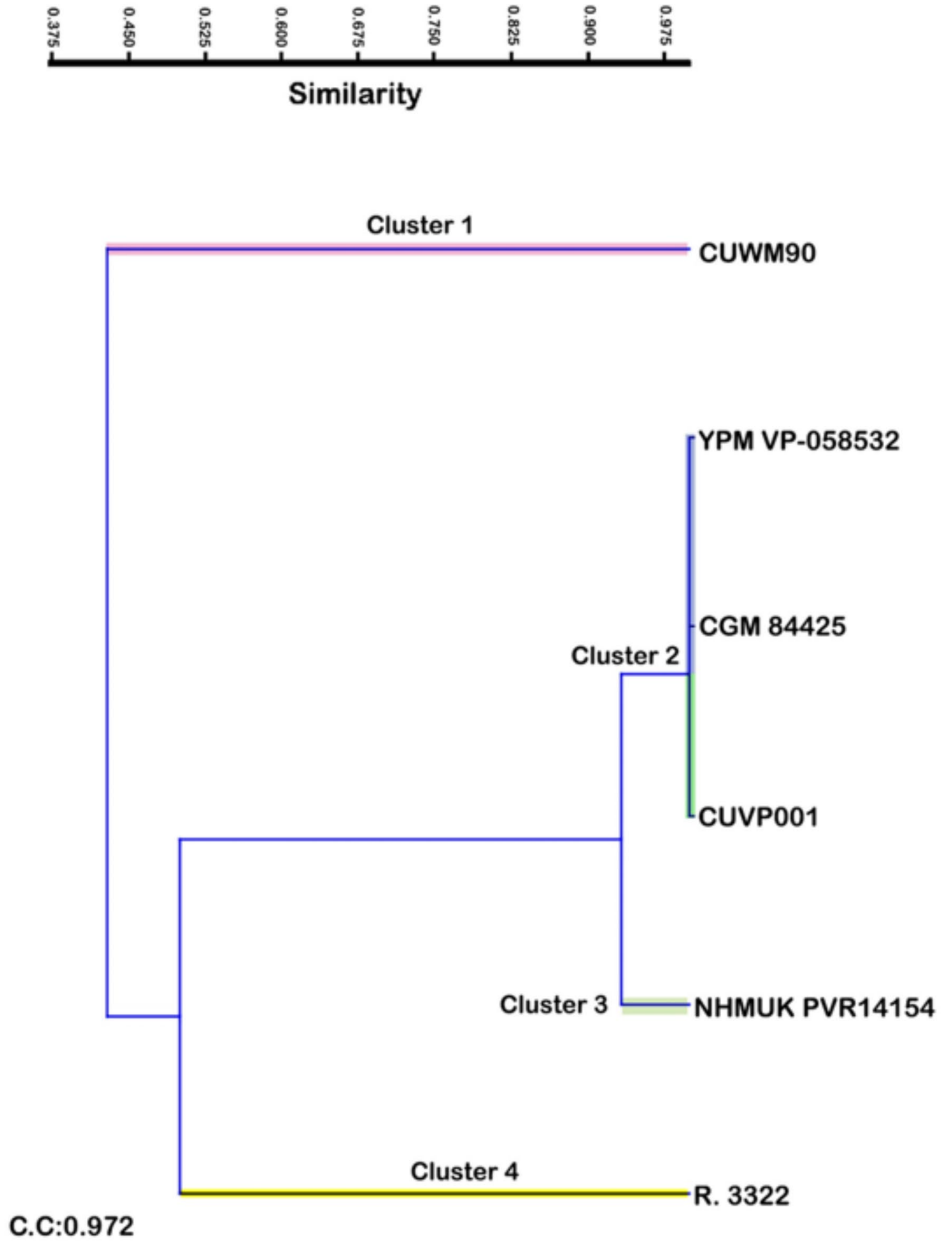
Cluster analysis of the morphometric measurements of different specimens. CUWM90, *Crocodylus sp.;* YPM VP-058532, *Crocodylus megarhinus*; CGM84425, *Crocodylus sp*.; CUVP001, *Crocodylus niloticus*; NHMUK PVR14154, *Rimasuchus lloydi*; NHMUK R. 3322, cast of *Crocodylus articeps*.

The cluster analysis (Fig. 12) of the different morphometric measurements mentioned in Table 1 (from A to P) for these specimens shows four clusters. Cluster number 1 includes only one species is which is less similar to *C. niloticus*. From the Eocene epoch, Cluster number 2 consists of three different species the first two are the most 2 species similar to each other almost up to 0.97. The third species is the recent crocodile which has a high degree of similarity to the first two species approximately 0.97. Cluster number 3 forms from only one species *Rimasuchus lloydi* which is similar to a cluster number 2. Cluster number 4 forms from only one species *Crocodylus articeps* which keeps similarity to the species in clusters number 2 and 3 but the closest one is *Rimasuchus lloydi*.

So, this analysis confirms parallel with the morphological studies that the closest species to the recent crocodile (*C. niloticus*) is FARB AMNH 5061, YPM VP-058532 which referred to *C. megrahinus* and CGM 84425 more than NHMUK PVR14154. Therefore, the most probably ancestor to the recent *C. niloticus* is *C. megrahinus* from the Eocene epoch more than *Rimasuchus lloydi* from Miocene epoch. Also, this cluster analysis demonstrate with morphological studies that the specimen of *Crocodylus sp*. CGM84425 is identical to both YPM VP-058532, FARB AMNH 5061 which referred to *C. megrahinus* except for a very few differences which may return to the maturity and health degree or the genus of each of them.

From the comparison, it suggests that the specimen of *Crocodylus* NHMUK R 3104 may be more closely related to NHMUK R 3322.

## Conclusion

This study demonstrated the high degree of differences in relation to the morphological features and dimensions of the cranial part of the living crocodile in Egypt which refers to *C. niloticus* and other broad-snouted extinct species from the same country over different epochs. Also, the present work showed the relationships between them. The Eocene epoch broad-snouted crocodile species are *C. articeps* and *C. megarhinus.* The Miocene epoch broad-snouted crocodile species are *R. lloydi* and *Crocodylus sp.* which preserves the broadest snout which is characteristic of the Miocene crocodilian. It confirmed by dimensions that however the *C. articeps* has a longer snout than the *C. megarhinus* of the closest snout breadth, *C. articeps* is a member of broad snouted crocodile rather than long snouted crocodile. The measurements and morphological characters showed that *C. articeps* and *C. megarhinus* are not the same species at different ages due to differences in maxillary alveoli number and shape as well as the differences in ratio between the snout width to snout length which indicates that *C. articeps* is more elongated than *C. megarhinus*. The *C. niloticus*,* C. articeps* and *C. megarhinus* differ from *R. lloydi* and *Crocodylus sp.* through measurements and morphology of them which proved that *R. lloydi* and *Crocodylus sp.* has the shape and proportions of an extreme brevirostrine crocodile this is exactly absent in the other mentioned species. It is found that both Eocene and Miocene crocodiles as well as *Rimasuchus lloydi* is closer in skull proportions of *C. niloticus*. But from the morphological characters it is shown that the *C. megarhinus* is the closest species and may a probable ancestor of *C. niloticus*. From the morphological features of the mandibles, it is deduced that *C. niloticus* lower jaw differs exactly from that of *Rimasuchus lloydi* and it is closer to that of both *C. megarhinus* and *C. articeps.*

From the morphometric and morphological results concerned to NHMUK PVR14154 it is concluded that the *Rimasuchus lloydi* is similar to a cluster number 2 and FARB AMNH 5061 but more similar especially to CGM84425. This indicates that the *Rimasuchus* contains the common characteristics of both *C. megrahinus* and *C. niloticus*.

From the cluster analysis and morphological results concerned to NHMUK R 3322 it is concluded that *C. articeps* contains unique characters related to it and doesn’t appear in other species beside this it contains a few similarities with *Rimasuchus lloydi*, FARB AMNH 5061, CGM84425 and *C. niloticus* respectively.

Egyptian Eocene *Crocodylus* is the ancestor to all known broad snouted species recorded from Egypt since the Eocene time. The closest species to the Eocene species is the living *Crocodylus niloticus*. That in fact make that most of the broad snouted crocodiles in Egypt are endemic.

Climatic change and a tectonic event may be the causes that led to the extinction of these species so, it is important to keep the only existing species in Lake Nasser and prevent the hunting of them to preserve the environmental biodiversity.

## Data Availability

All data generated and / or analyzed during this study are included in this article.

## Declarations

### Statements

The present specimen of *Crocodylus niloticus* was collected near Lake Nasser under an ethical reference with approval number CU I F C 38 23 issued by Cairo University’s Institutional Animal Care & Use Committee CU-LACUC. This specimen is a mature female of a total length of 218 cm. The skull is complete with a length measuring 34 cm. CUVP001 is referred to the upper and lower jaw of *C. niloticus* which is preserved in CUVP.
